# Supplementary material for: Risk of long COVID and associated symptoms after acute SARS-COV-2 infection in ethnic minorities: A nationwide register-linked cohort study in Denmark
Source: PLoS Med. 2024 Feb 20;21(2):e1004280. doi: 10.1371/journal.pmed.1004280 (PMC10914299; doi:10.1371/journal.pmed.1004280)
Supplement: S1 Table — COVID-19, Coronavirus Disease 2019. (DOCX) [file pmed.1004280.s001.docx]

**S1 Table. List of International Classification of Diseases (ICD-10) codes included.**

| **Symptom** | **ICD-10 codes** |
| --- | --- |
| Fatigue | R53, R539, R539A, R539C, R539E, R539F, G933 |
| Headache | R51–­­­­R519 |
| Dyspnoea (difficulty in breathing) | R06, R060, R060A, R061, R063, R064, R065, R066, R068, R068A, R068B, R068C, R068D, R068E |
| Cough | R05–R059 |
| Chest pain | R07–R074 |
| Depression/anxiety | F32–F339, F411–F418, F430–F4300 |
| **Diagnosis** |  |
| COVID-19 | B34.2, B34.2A, B97.2, B97.2A |
| Long COVID | B94.8, B94.8A |
| Myocardial infarction | I21–I21.9, I22, I25.2 |
| Congestive heart failure | I10–I10.9, I11, I11.0, I13.0, I13.2, I25.5, I42.0, I42.6, I42.7, I42.8, I42.9, I43, I50, I50.0, I50.1, I50.9 |
| Peripheral vascular disease | I70, 171, I73.1, I73.8, I73.9, I77.1, I79.0, I79.2 |
| Cerebrovascular disease | G45, I60–I64, I67, I69 |
| Chronic obstructive pulmonary disease | J43, J44–J44.9 |
| Rheumatic disease | M05, M06, M12.3, M07.0–M07.3 |
| Dementia | F00–F03, F05.1, G30, G31.1, G31.9 |
| Peptic ulcer disease | K25–K28 |
| Hemiplegia | G11.4, G80, G81, G82, G83.0–G83.3, G83.8 |
| Diabetes without complications | E10, E11, E10.0, E10.0, E10.1, E11.0, E11.1, E12.0, E12.1, E13.0, E13.1, E14.0, E14.1 |
| Diabetes with complications | E10, E11, E10.2, E10.5, E10.7, E11.2, E11.7, E12.2, E12.7, E13.2, E13.7, E14.2, E14.7 |
| Mild liver disease | B15–B19, K70, K70.0, K70.1 |
| Moderate to severe liver disease | I85.0, I85.9, I98.2, I98.3, K70.3, K70.4, K70.9, K73, K74.0, K74.6, K75.4 |
| Renal disease | I12.0, I13.1, N03.2–N03.7, N05.2–N05.7, N11, N18, N19, N25.0, Q61.1, Z49, Z94.0, Z99.2 |
| Malignancy | C00–C09, C10–C41, C45–C58, C60–C76, C81–C86, C88–C97 |
| Metastatic cancer | C77–C80 |
| Acquired immunodeficiency syndrome | B20–B24, R75, Z21 |

COVID-19=coronavirus disease 2019.
