# Supplementary material for: Risk of long COVID and associated symptoms after acute SARS-COV-2 infection in ethnic minorities: A nationwide register-linked cohort study in Denmark
Source: PLoS Med. 2024 Feb 20;21(2):e1004280. doi: 10.1371/journal.pmed.1004280 (PMC10914299; doi:10.1371/journal.pmed.1004280)
Supplement: S2 Table — Data are in median (IQR) or n (%). *Family income was the total household disposable income among patients with COVID-19 in the specific calendar year. §CCI composed myocardial infarction, congestive heart failure, peripheral vascular disease, cerebrovascular disease, chronic obstructive pulmonary disease, rheumatic disease, dementia, peptic ulcer disease, hemiplegia, diabetes without complications, diabetes with complications, mild liver disease, moderate to severe liver disease, renal disease, malignancy, metastatic cancer, and AIDS. AIDS, acquired immunodeficiency syndrome; CCI, Charlson comorbidity index; COVID-19, Coronavirus Disease 2019; IQR, interquartile range; NA, not applicable; SARS-CoV-2, Severe Acute Respiratory Syndrome Coronavirus 2. (DOCX) [file pmed.1004280.s002.docx]

**S2 Table. Individuals who had first-time tested positive for SARS-COV-2 between January 2020 and August 2022 by largest countries of origin.**

|  | **Denmark** | **Norway** | **Sweden** | **Afghanistan** | **Iraq** | **Iran** | **Somalia** | **Pakistan** | **Turkey** |
| --- | --- | --- | --- | --- | --- | --- | --- | --- | --- |
| **n** | 1 952 021 | 7200 | 7078 | 9273 | 15 600 | 9200 | 9077 | 12 462 | 35 460 |
| **Immigrants** | NA | 6680 (92.8%) | 6395 (90.4%) | 8455 (91.2%) | 12 571 (80.1%) | 7667 (83.3%) | 6274 (69.1%) | 7031 (56.4%) | 18 764 (52.9%) |
| **Descendants** | NA | 520 (7.2%) | 683 (9.6%) | 818 (8.8%) | 3119 (19.9%) | 1533 (16.7%) | 2803 (30.9%) | 5431 (43.6%) | 16 696 (47.1%) |
| **Length of residency, years** | NA | 36 (17–41) | 36 (16–38) | 19 (16–21) | 22 (19–26) | 27 (14–34) | 24 (20–27) | 36 (29–39) | 34 (26–38) |
| **Age, years** | 61 (43–75) | 62 (39–76) | 64 (41–76) | 44 (32–58) | 48 (32–60) | 49 (36–60) | 43 (28–56) | 56 (42–71) | 49 (36–62) |
| **Sex** |  |  |  |  |  |  |  |  |  |
| Female | 1 026 373 (52.6%) | 4752 (66.0%) | 4308 (60.8%) | 4470 (48.2%) | 7807 (49.8%) | 4400 (47.8%) | 4856 (53.4%) | 6415 (51.5%) | 18 358 (51.8%) |
| Male | 925 648 (47.4%) | 2448 (34.0%) | 2770 (39.2%) | 4803 (51.8%) | 7793 (50.2%) | 4800 (52.2%) | 4221 (46.6%) | 6047 (48.5%) | 17 102 (48.2%) |
| **Civil status** | | | | | | | | | |
| Cohabiting | 862 240 (44.2%) | 2615 (36.3%) | 2903 (41.0%) | 4211 (45.4%) | 6402 (40.8%) | 4126 (44.8%) | 2030 (22.4%) | 7832 (62.8%) | 19 954 (56.3%) |
| Living alone | 852 792 (43.7%) | 3824 (53.1%) | 3464 (48.9%) | 4610 (49.7%) | 7730 (49.3%) | 3832 (41.7%) | 5894 (64.9%) | 3672 (29.5%) | 11 723 (33.0%) |
| Other | 236 989 (12.1%) | 761 (10.6%) | 711 (10.1%) | 452 (4.9%) | 1558 (9.9%) | 1242 (13.5%) | 1153 (12.7%) | 958 (7.7%) | 3783 (10.7%) |
| **Education** | | | | | | | | | |
| Low | 465 063 (23.8%) | 597 (8.3%) | 663 (9.4%) | 3363 (36.3%) | 6399 (40.8%) | 2365 (25.7%) | 4684 (51.6%) | 4426 (35.5%) | 15 902 (44.8%) |
| Medium | 904 288 (46.3%) | 2464 (34.2%) | 2406 (34.0%) | 3183 (34.3%) | 5242 (33.4%) | 3060 (33.3%) | 2724 (30.0%) | 4086 (32.8%) | 11 904 (33.6%) |
| High | 571 393 (29.3%) | 3769 (52.4%) | 3522 (49.7%) | 1581 (17.0%) | 2728 (17.4%) | 3309 (36.0%) | 682 (7.5%) | 3293 (26.4%) | 5304 (15.0%) |
| Missing | 11 277 (0.6%) | 370 (5.1%) | 487 (6.9%) | 1146 (12.4%) | 1321 (8.4%) | 466 (5.0%) | 987 (10.9%) | 657 (5.3%) | 2350 (6.6%) |
| **Family income*** | | | | | | | | | |
| Low | 353 452 (18.1%) | 2441 (33.9%) | 1877 (26.5%) | 5783 (62.4%) | 9801 (62.5%) | 4073 (44.3%) | 6921 (76.3%) | 6437 (51.7%) | 16 345 (46.1%) |
| Middle | 585 134 (30.0%) | 1423 (19.8%) | 1541 (21.8%) | 1896 (20.4%) | 2845 (18.1%) | 2042 (22.2%) | 1108 (12.2%) | 3243 (26.0%) | 11 055 (31.2%) |
| High | 859 757 (44.0%) | 2728 (37.9%) | 3160 (44.6%) | 839 (9.1%) | 1571 (10.0%) | 2279 (24.8%) | 249 (2.7%) | 1904 (15.3%) | 5632 (15.9%) |
| Missing | 153 678 (7.9%) | 608 (8.4%) | 500 (7.1%) | 755 (8.1%) | 1473 (9.4%) | 806 (8.7%) | 799 (8.8%) | 878 (7.0%) | 2428 (6.8%) |
| **COVID-19 hospitalisation** | 30 230 (1.5%) | 123 (1.7%) | 142 (2.0%) | 370 (3.9%) | 831 (5.1%) | 366 (3.9%) | 306 (3.3%) | 618 (4.8%) | 1547 (4.3%) |
| **Intensive care** | 12 014 (0.6%) | 35 (0.5%) | 37 (0.5%) | 28 (0.3%) | 78 (0.5%) | 36 (0.4%) | 68 (0.7%) | 74 (0.6%) | 160 (0.4%) |
| **COVID-19 vaccination** | | | | | | | | | |
| One dose | 1 813 312 (92.9%) | 6320 (87.8%) | 6258 (88.4%) | 7565 (81.5%) | 11 037 (70.3%) | 7859 (85.4%) | 5713 (62.9%) | 9957 (79.9%) | 23 754 (67.0%) |
| Two doses | 1 796 381 (92.0%) | 6199 (86.1%) | 6184 (87.4%) | 7246 (78.1%) | 10 482 (66.8%) | 7689 (83.6%) | 5248 (57.8%) | 9586 (76.9%) | 22 559 (63.6%) |
| Three doses | 1 489 444 (76.3%) | 4889 (67.9%) | 4856 (68.6%) | 3667 (39.5%) | 4684 (29.8%) | 5278 (57.3%) | 1586 (17.5%) | 4523 (36.3%) | 10 760 (30.3%) |
| **Charlson comorbidity index**^§^ | | | | | | | | | |
| 0 | 1 550 412 (79.4%) | 5971 (82.9%) | 5789 (81.8%) | 6974 (75.2%) | 11 356 (72.4%) | 6774 (73.6%) | 7179 (79.1%) | 8900 (71.4%) | 25 568 (72.1%) |
| 1–2 | 396 200 (20.3%) | 1210 (16.8%) | 1274 (18.0%) | 2291 (24.7%) | 4318 (27.5%) | 2410 (26.2%) | 1884 (20.8%) | 3549 (28.5%) | 9856 (27.8%) |
| ≥3 | 5409 (0.3%) | 19 (0.3%) | 15 (0.2%) | 8 (0.1%) | 16 (0.1%) | 16 (0.2%) | 14 (0.1%) | 13 (0.1%) | 36 (0.1%) |

Data are in median (IQR) or n (%). *Family income (presented in tertiles) was the total household disposable income among patients with COVID-19 in the specific calendar year. ^§^Charlson comorbidity index composed myocardial infarction, congestive heart failure, peripheral vascular disease, cerebrovascular disease, chronic obstructive pulmonary disease, rheumatic disease, dementia, peptic ulcer disease, hemiplegia, diabetes without complications, diabetes with complications, mild liver disease, moderate to severe liver disease, renal disease, malignancy, metastatic cancer, and acquired immunodeficiency syndrome (AIDS). SARS-COV-2=severe acute respiratory syndrome coronavirus 2. COVID-19=coronavirus disease 2019. IQR=interquartile range. NA=not applicable.
