# Supplementary material for: Risk of long COVID and associated symptoms after acute SARS-COV-2 infection in ethnic minorities: A nationwide register-linked cohort study in Denmark
Source: PLoS Med. 2024 Feb 20;21(2):e1004280. doi: 10.1371/journal.pmed.1004280 (PMC10914299; doi:10.1371/journal.pmed.1004280)
Supplement: S3 Table — Northern Europe indicates Northern Europe other than Denmark. *Standardised to 2020 Danish population age distribution. CI, confidence interval; IR, incidence rate. (DOCX) [file pmed.1004280.s003.docx]

**S3 Table. Age-standardised incidence rates of long COVID per 100000 person-years by region of origin.**

|  | **n** | **IR***  **(95% CI)** |
| --- | --- | --- |
| Denmark | 3468 | 43 (42 to 45) |
| Northern Europe | 47 | 58 (42 to 78) |
| Western Europe | 45 | 32 (23 to 43) |
| Eastern Europe | 373 | 121 (106 to 137) |
| Asia | 204 | 131 (110 to 155) |
| Middle East | 312 | 228 (198 to 262) |
| North Africa | 62 | 253 (189 to 335) |
| Subsaharan Africa | 68 | 163 (115 to 227) |

Northern Europe indicates Northern Europe other than Denmark. *Standardised to 2020 Danish population age distribution. IR=incidence rate. CI=confidence interval.
