# Supplementary material for: Risk of long COVID and associated symptoms after acute SARS-COV-2 infection in ethnic minorities: A nationwide register-linked cohort study in Denmark
Source: PLoS Med. 2024 Feb 20;21(2):e1004280. doi: 10.1371/journal.pmed.1004280 (PMC10914299; doi:10.1371/journal.pmed.1004280)
Supplement: S4 Table — Northern Europe indicates Northern Europe other than Denmark. The adjusted model composed age, sex, civil status, education, family income, and CCI. CCI, Charlson comorbidity index; CI, confidence interval; HR, hazard ratio. (DOCX) [file pmed.1004280.s004.docx]

**S4 Table. Hazard ratios of long COVID diagnosis among individuals aged 18–60 years and >60 years by region of origin.**

|  | **18–60 years** | | | **>60 years** | | |
| --- | --- | --- | --- | --- | --- | --- |
|  | **n** | **Unadjusted**  **HR (95% CI)** | **Adjusted**  **HR (95% CI)** | **n** | **Unadjusted**  **HR (95% CI)** | **Adjusted**  **HR (95% CI)** |
| Denmark | 2126 | 1.00 (reference) | 1.00 (reference) | 1342 | 1.00 (reference) | 1.00 (reference) |
| Northern Europe | 32 | 1.08 (0.79 to 1.48) | 1.44 (1.05 to 1.98) | 15 | 0.65 (0.43 to 0.99) | 0.64 (0.42 to 0.99) |
| Western Europe | 26 | 0.59 (0.42 to 0.83) | 0.74 (0.52 to 1.06) | 19 | 0.82 (0.57 to 1.18) | 0.90 (0.62 to 1.30) |
| Eastern Europe | 293 | 1.04 (0.93 to 1.15) | 1.19 (1.06 to 1.34) | 80 | 1.50 (1.28 to 1.77) | 1.62 (1.35 to 1.97) |
| Asia | 150 | 0.95 (0.83 to 1.10) | 0.93 (0.80 to 1.09) | 54 | 1.49 (1.21 to 1.82) | 2.12 (1.72 to 2.60) |
| Middle East | 243 | 1.10 (0.99 to 1.24) | 1.16 (1.03 to 1.32) | 69 | 1.73 (1.41 to 2.11) | 1.98 (1.57 to 2.50) |
| North Africa | 47 | 1.39 (1.08 to 1.77) | 1.38 (1.06 to 1.80) | 15 | 1.09 (0.72 to 1.65) | 1.15 (0.69 to 1.92) |
| Subsaharan Africa | 53 | 0.67 (0.52 to 0.86) | 0.85 (0.64 to 1.12) | 15 | 2.10 (1.45 to 3.03) | 3.27 (2.24 to 4.79) |

Northern Europe indicates Northern Europe other than Denmark. The adjusted model composed age, sex, civil status, education, family income, and Charlson comorbidity index. HR=hazard ratio. CI=confidence interval.
