# Supplementary material for: Risk of long COVID and associated symptoms after acute SARS-COV-2 infection in ethnic minorities: A nationwide register-linked cohort study in Denmark
Source: PLoS Med. 2024 Feb 20;21(2):e1004280. doi: 10.1371/journal.pmed.1004280 (PMC10914299; doi:10.1371/journal.pmed.1004280)
Supplement: S5 Table — Northern Europe indicates Northern Europe other than Denmark. The adjusted model composed age, sex, civil status, education, family income, and CCI. CCI, Charlson comorbidity index; CI, confidence interval; HR, hazard ratio. (DOCX) [file pmed.1004280.s005.docx]

**S5 Table. Hazard ratios of long COVID diagnosis by age group.**

|  | **Age groups**  **(years)** | **n** | **Unadjusted**  **HR (95% CI)** | **Adjusted**  **HR (95% CI)** |
| --- | --- | --- | --- | --- |
| Denmark | 18–60 | 2126 | 1.00 (reference) | 1.00 (reference) |
|  | >60 | 1342 | 0.76 (0.72 to 0.80) | 0.52 (0.49 to 0.55) |
| Northern Europe | 18–60 | 32 | 1.08 (0.79 to 1.48) | 1.44 (1.05 to 1.98) |
|  | >60 | 15 | 0.50 (0.33 to 0.75) | 0.34 (0.22 to 0.52) |
| Western Europe | 18–60 | 26 | 0.59 (0.42 to 0.83) | 0.74 (0.52 to 1.06) |
|  | >60 | 19 | 0.62 (0.43 to 0.90) | 0.47 (0.33 to 0.68) |
| Eastern Europe | 18–60 | 293 | 1.04 (0.93 to 1.15) | 1.19 (1.06 to 1.33) |
|  | >60 | 80 | 1.15 (0.97 to 1.35) | 0.85 (0.70 to 1.04) |
| Asia | 18–60 | 150 | 0.95 (0.83 to 1.10) | 0.93 (0.79 to 1.08) |
|  | >60 | 54 | 1.13 (0.92 to 1.38) | 1.11 (0.90 to 1.37) |
| Middle East | 18–60 | 243 | 1.10 (0.99 to 1.24) | 1.16 (1.02 to 1.32) |
|  | >60 | 69 | 1.31 (1.07 to 1.61) | 1.04 (0.83 to 1.32) |
| North Africa | 18–60 | 47 | 1.38 (1.08 to 1.77) | 1.38 (1.06 to 1.79) |
|  | >60 | 15 | 0.83 (0.55 to 1.26) | 0.60 (0.36 to 1.01) |
| Subsaharan Africa | 18–60 | 53 | 0.67 (0.52 to 0.86) | 0.85 (0.64 to 1.12) |
|  | >60 | 15 | 1.60 (1.11 to 2.31) | 1.72 (1.17 to 2.52) |

Northern Europe indicates Northern Europe other than Denmark. The adjusted model composed age, sex, civil status, education, family income, and Charlson comorbidity index. HR=hazard ratio. CI=confidence interval.
