# Supplementary material for: Risk of long COVID and associated symptoms after acute SARS-COV-2 infection in ethnic minorities: A nationwide register-linked cohort study in Denmark
Source: PLoS Med. 2024 Feb 20;21(2):e1004280. doi: 10.1371/journal.pmed.1004280 (PMC10914299; doi:10.1371/journal.pmed.1004280)
Supplement: S6 Table — Northern Europe indicates Northern Europe other than Denmark. The adjusted model composed age, sex, civil status, education, family income, and CCI. CCI, Charlson comorbidity index; CI, confidence interval; HR, hazard ratio. (DOCX) [file pmed.1004280.s006.docx]

**S6 Table. Hazard ratios of long COVID diagnosis by sex.**

|  | **Sex** | **n** | **Adjusted**  **HR (95% CI)** |
| --- | --- | --- | --- |
| Denmark | Female | 1999 | 1.00 (reference) |
|  | Male | 1469 | 0.86 (0.82 to 0.91) |
| Northern Europe | Female | 35 | 1.00 (reference) |
|  | Male | 12 | 0.57 (0.32 to 1.00) |
| Western Europe | Female | 14 | 1.00 (reference) |
|  | Male | 31 | 1.81 (1.05 to 3.13) |
| Eastern Europe | Female | 192 | 1.00 (reference) |
|  | Male | 181 | 1.49 (1.24 to 1.79) |
| Asia | Female | 114 | 1.00 (reference) |
|  | Male | 90 | 1.03 (0.81 to 1.31) |
| Middle East | Female | 148 | 1.00 (reference) |
|  | Male | 164 | 1.74 (1.41 to 2.14) |
| North Africa | Female | 37 | 1.00 (reference) |
|  | Male | 25 | 1.17 (0.73 to 1.86) |
| Subsaharan Africa | Female | 35 | 1.00 (reference) |
|  | Male | 33 | 1.88 (1.19 to 2.95) |

Northern Europe indicates Northern Europe other than Denmark. The adjusted model composed age, sex, civil status, education, family income, and Charlson comorbidity index. HR=hazard ratio. CI=confidence interval.
