# Supplementary material for: Risk of long COVID and associated symptoms after acute SARS-COV-2 infection in ethnic minorities: A nationwide register-linked cohort study in Denmark
Source: PLoS Med. 2024 Feb 20;21(2):e1004280. doi: 10.1371/journal.pmed.1004280 (PMC10914299; doi:10.1371/journal.pmed.1004280)
Supplement: S7 Table — Northern Europe indicates Northern Europe other than Denmark. The adjusted model composed age, sex, civil status, education, family income, and CCI. CCI, Charlson comorbidity index; CI, confidence interval; HR, hazard ratio. (DOCX) [file pmed.1004280.s007.docx]

**S7 Table. Hazard ratios of long COVID diagnosis for males and females by region of origin.**

|  |  | **Male** |  | **Female** |
| --- | --- | --- | --- | --- |
|  | **n** | **Adjusted**  **HR (95% CI)** | **n** | **Adjusted**  **HR (95% CI)** |
| Denmark | 1469 | 1.00 (reference) | 1999 | 1.00 (reference) |
| Northern Europe | 12 | 0.73 (0.45 to 1.17) | 35 | 1.10 (0.81 to 1.49) |
| Western Europe | 31 | 1.11 (0.81 to 1.51) | 14 | 0.53 (0.33 to 1.83) |
| Eastern Europe | 181 | 1.81 (1.58 to 2.08) | 192 | 1.05 (0.92 to 1.21) |
| Asia | 90 | 1.35 (1.12 to 1.62) | 114 | 1.13 (0.95 to 1.34) |
| Middle East | 164 | 1.94 (1.68 to 2.24) | 148 | 0.97 (0.81 to 1.15) |
| North Africa | 25 | 1.68 (1.18 to 2.38) | 37 | 1.24 (0.91 to 1.69) |
| Subsaharan Africa | 33 | 1.81 (1.35 to 2.43) | 35 | 0.83 (0.59 to 1.19) |

Northern Europe indicates Northern Europe other than Denmark. The adjusted model composed age, sex, civil status, education, family income, and Charlson comorbidity index. HR=hazard ratio. CI=confidence interval.
