# Supplementary material for: Risk of long COVID and associated symptoms after acute SARS-COV-2 infection in ethnic minorities: A nationwide register-linked cohort study in Denmark
Source: PLoS Med. 2024 Feb 20;21(2):e1004280. doi: 10.1371/journal.pmed.1004280 (PMC10914299; doi:10.1371/journal.pmed.1004280)
Supplement: S8 Table — Northern Europe indicates Northern Europe other than Denmark. The adjusted model composed age, sex, civil status, education, family income, and CCI. CCI, Charlson comorbidity index; CI, confidence interval; HR, hazard ratio. (DOCX) [file pmed.1004280.s008.docx]

**S8 Table. Hazard ratios of long COVID diagnosis among hospitalised and non-hospitalised individuals by region of origin.**

|  | **Hospitalised** | | | **Non-Hospitalised** | | |
| --- | --- | --- | --- | --- | --- | --- |
|  | **n** | **Unadjusted**  **HR (95% CI)** | **Adjusted**  **HR (95% CI)** | **n** | **Unadjusted**  **HR (95% CI)** | **Adjusted**  **HR (95% CI)** |
| Denmark | 1483 | 1.00 (reference) | 1.00 (reference) | 1985 | 1.00 (reference) | 1.00 (reference) |
| Northern Europe | 22 | 1.15 (0.76 to 1.76) | 1.21 (0.78 to 1.89) | 25 | 0.79 (0.58 to 1.07) | 0.90 (0.66 to 1.23) |
| Western Europe | 23 | 0.94 (0.62 to 1.42) | 0.91 (0.59 to 1.39) | 22 | 0.59 (0.43 to 0.81) | 0.73 (0.53 to 1.01) |
| Eastern Europe | 204 | 1.63 (1.41 to 1.89) | 1.59 (1.35 to 1.86) | 169 | 1.05 (0.94 to 1.17) | 1.15 (1.02 to 1.30) |
| Asia | 108 | 1.35 (1.11 to 1.64) | 1.22 (0.98 to 1.50) | 96 | 1.04 (0.90 to 1.20) | 1.14 (0.98 to 1.33) |
| Middle East | 161 | 1.55 (1.32 to 1.83) | 1.57 (1.31 to 1.88) | 151 | 1.14 (1.01 to 1.29) | 1.21 (1.05 to 1.39) |
| North Africa | 33 | 1.27 (0.90 to 1.79) | 1.41 (1.02 to 2.04) | 29 | 1.25 (0.96 to 1.63) | 1.27 (0.94 to 1.71) |
| Subsaharan Africa | 37 | 1.29 (0.93 to 1.79) | 1.52 (1.07 to 2.15) | 31 | 0.77 (0.58 to 1.01) | 0.99 (0.73 to 1.33) |

Northern Europe indicates Northern Europe other than Denmark. The adjusted model composed age, sex, civil status, education, family income, and Charlson comorbidity index. HR=hazard ratio. CI=confidence interval.
