# Supplementary material for: Risk of long COVID and associated symptoms after acute SARS-COV-2 infection in ethnic minorities: A nationwide register-linked cohort study in Denmark
Source: PLoS Med. 2024 Feb 20;21(2):e1004280. doi: 10.1371/journal.pmed.1004280 (PMC10914299; doi:10.1371/journal.pmed.1004280)
Supplement: S9 Table — Northern Europe indicates Northern Europe other than Denmark. *Estimates are not displayed due to small numbers in accordance with Danish Data Protection Act. The adjusted model composed age, sex, civil status, education, family income, and CCI. CCI, Charlson comorbidity index; CI, confidence interval; HR, hazard ratio. (DOCX) [file pmed.1004280.s009.docx]

**S9 Table. Hazard ratios of long COVID diagnosis by number of doses of COVID-19 vaccine.**

Northern Europe indicates Northern Europe other than Denmark. *Estimates are not displayed due to small numbers in accordance with Danish Data Protection Act. The adjusted model composed age, sex, civil status, education, family income, and Charlson comorbidity index. HR=hazard ratio. CI=confidence interval.

|  | **Unvaccinated** | | **One dose** | | **Two doses** | | **Three doses** | |
| --- | --- | --- | --- | --- | --- | --- | --- | --- |
|  | **n** | **Adjusted**  **HR (95% CI)** | **n** | **Adjusted**  **HR (95% CI)** | **n** | **Adjusted**  **HR (95% CI)** | **n** | **Adjusted**  **HR (95% CI)** |
| Denmark | 203 | 1.00 (reference) | 34 | 0.79 (0.59 to 1.06) | 316 | 0.53 (0.46 to 0.61) | 2915 | 0.68 (0.61 to 0.76) |
| Northern Europe | * | 1.00 (reference) | * | * | 10 | 3.23 (0.73 to 14.10) | 35 | 1.50 (0.36 to 6.22) |
| Western Europe | * | 1.00 (reference) | * | * | 6 | 0.74 (0.21 to 2.55) | 34 | 0.91 (0.36 to 2.29) |
| Eastern Europe | 98 | 1.00 (reference) | 19 | 1.91 (1.18 to 3.07) | 101 | 1.01 (0.78 to 1.31) | 155 | 0.96 (0.76 to 1.21) |
| Asia | 16 | 1.00 (reference) | 6 | 1.42 (0.33 to 6.01) | 42 | 1.60 (0.95 to 2.68) | 140 | 1.94 (1.21 to 3.11) |
| Middle East | 51 | 1.00 (reference) | 7 | 1.18 (0.96 to 1.44) | 114 | 1.80 (1.31 to 2.47) | 140 | 1.74 (1.28 to 2.36) |
| North Africa | 23 | 1.00 (reference) | 5 | 0.80 (0.10 to 5.97) | 15 | 1.12 (0.64 to 1.96) | 19 | 0.70 (0.39 to 1.25) |
| Subsaharan Africa | 13 | 1.00 (reference) | 5 | 0.90 (0.20 to 4.01) | 16 | 1.12 (0.55 to 2.25) | 34 | 1.84 (0.99 to 3.43) |
