# Supplementary material for: Risk of long COVID and associated symptoms after acute SARS-COV-2 infection in ethnic minorities: A nationwide register-linked cohort study in Denmark
Source: PLoS Med. 2024 Feb 20;21(2):e1004280. doi: 10.1371/journal.pmed.1004280 (PMC10914299; doi:10.1371/journal.pmed.1004280)
Supplement: S10 Table — Northern Europe indicates Northern Europe other than Denmark. Hospital contacts related to cardiopulmonary symptoms included dyspnoea (difficulty in breathing), cough, and chest pain as a composite outcome. Hospital contacts related to any long COVID symptoms included fatigue, headache, dyspnoea (difficulty in breathing), cough, chest pain, depression, and/or anxiety as a composite outcome. The adjusted model composed age, sex, civil status, education, family income, and CCI. CCI, Charlson comorbidity index; CI, confidence interval; COVID-19, Coronavirus Disease 2019; OR, odds ratio. (DOCX) [file pmed.1004280.s010.docx]

**S10 Table. Odds ratio of hospital contacts related to specific symptoms 6 months after COVID-19 diagnosis compared with 6 months before COVID-19 diagnosis by region of origin.**

|  | **6 months before COVID-19 diagnosis** | **6 months after COVID-19 diagnosis** | |
| --- | --- | --- | --- |
|  | **OR (95% CI)** | **Unadjusted**  **OR (95% CI)** | **Adjusted**  **OR (95% CI)** |
| **Hospital contacts related to fatigue** | | | |
| Denmark | 1.00 (reference) | 3.10 (2.96 to 3.25) | 3.06 (2.91 to 3.21) |
| Northern Europe | 1.00 (reference) | 4.41 (2.84 to 6.84) | 4.21 (2.64 to 6.71) |
| Western Europe | 1.00 (reference) | 2.93 (1.99 to 4.31) | 3.31 (2.20 to 4.97) |
| Eastern Europe | 1.00 (reference) | 2.76 (2.27 to 3.36) | 2.62 (2.10 to 3.27) |
| Asia | 1.00 (reference) | 2.62 (1.94 to 3.54) | 2.70 (1.94 to 3.76) |
| Middle East | 1.00 (reference) | 2.28 (1.81 to 2.87) | 2.49 (1.93 to 3.21) |
| North Africa | 1.00 (reference) | 1.07 (0.54 to 2.09) | 0.98 (0.46 to 2.10) |
| Subsaharan Africa | 1.00 (reference) | 2.95 (1.90 to 4.58) | 4.89 (2.90 to 8.24) |
| **Hospital contacts related to headache** | | | |
| Denmark | 1.00 (reference) | 3.30 (3.16 to 3.45) | 3.30 (3.14 to 3.46) |
| Northern Europe | 1.00 (reference) | 3.05 (2.04 to 4.55) | 3.65 (2.34 to 5.70) |
| Western Europe | 1.00 (reference) | 3.91 (2.59 to 5.88) | 3.50 (2.24 to 5.46) |
| Eastern Europe | 1.00 (reference) | 2.63 (2.29 to 3.03) | 2.84 (2.43 to 3.33) |
| Asia | 1.00 (reference) | 2.65 (2.13 to 3.29) | 2.58 (2.02 to 3.29) |
| Middle East | 1.00 (reference) | 2.11 (1.78 to 2.51) | 2.34 (1.93 to 2.84) |
| North Africa | 1.00 (reference) | 1.36 (0.91 to 2.02) | 1.60 (1.03 to 2.50) |
| Subsaharan Africa | 1.00 (reference) | 1.88 (1.36 to 2.59) | 2.00 (1.41 to 2.84) |
| **Hospital contacts related to cardiopulmonary symptoms** | | | |
| Denmark | 1.00 (reference) | 3.97 (3.91 to 4.04) | 4.10 (4.03 to 4.17) |
| Northern Europe | 1.00 (reference) | 3.68 (3.10 to 4.37) | 4.45 (3.68 to 5.37) |
| Western Europe | 1.00 (reference) | 3.40 (2.96 to 3.90) | 3.55 (3.06 to 4.12) |
| Eastern Europe | 1.00 (reference) | 4.00 (3.76 to 4.26) | 4.47 (4.17 to 4.80) |
| Asia | 1.00 (reference) | 3.51 (3.21 to 3.83) | 3.69 (3.35 to 4.07) |
| Middle East | 1.00 (reference) | 3.57 (3.31 to 3.83) | 4.31 (3.96 to 4.68) |
| North Africa | 1.00 (reference) | 3.99 (3.31 to 4.82) | 4.68 (3.81 to 5.74) |
| Subsaharan Africa | 1.00 (reference) | 2.68 (2.27 to 3.16) | 3.38 (2.81 to 4.07) |
| **Hospital contacts related to any long COVID symptoms** | | | |
| Denmark | 1.00 (reference) | 4.14 (4.09 to 4.19) | 4.30 (4.25 to 4.36) |
| Northern Europe | 1.00 (reference) | 3.78 (3.33 to 4.28) | 4.36 (3.80 to 5.01) |
| Western Europe | 1.00 (reference) | 3.73 (3.37 to 4.14) | 3.92 (3.51 to 4.38) |
| Eastern Europe | 1.00 (reference) | 3.71 (3.54 to 3.89) | 4.15 (3.93 to 4.38) |
| Asia | 1.00 (reference) | 3.60 (3.36 to 3.85) | 3.71 (3.44 to 4.01) |
| Middle East | 1.00 (reference) | 3.24 (3.07 to 3.43) | 3.83 (3.59 to 4.08) |
| North Africa | 1.00 (reference) | 3.37 (2.93 to 3.86) | 4.07 (3.49 to 4.73) |
| Subsaharan Africa | 1.00 (reference) | 2.73 (2.41 to 3.09) | 3.29 (2.86 to 3.77) |

Northern Europe indicates Northern Europe other than Denmark. Hospital contacts related to cardiopulmonary symptoms included dyspnoea (difficulty in breathing), cough, and chest pain as a composite outcome. Hospital contacts related to any long COVID symptoms included fatigue, headache, dyspnoea (difficulty in breathing), cough, chest pain, depression and/or anxiety as a composite outcome. The adjusted model composed age, sex, civil status, education, family income, and Charlson comorbidity index. OR=odds ratio. CI=confidence interval.
