# Supplementary material for: Risk of long COVID and associated symptoms after acute SARS-COV-2 infection in ethnic minorities: A nationwide register-linked cohort study in Denmark
Source: PLoS Med. 2024 Feb 20;21(2):e1004280. doi: 10.1371/journal.pmed.1004280 (PMC10914299; doi:10.1371/journal.pmed.1004280)
Supplement: S11 Table — Hospital contacts related to any long COVID symptoms included fatigue, headache, dyspnoea (difficulty in breathing), cough, chest pain, depression and/or anxiety as a composite outcome. The adjusted model composed age, sex, civil status, education, family income, and CCI. CCI, Charlson comorbidity index; CI, confidence interval; COVID-19, Coronavirus Disease 2019; OR, odds ratio. (DOCX) [file pmed.1004280.s011.docx]

**S11 Table. Odds ratio of hospital contacts related to any long COVID symptoms 6 months after COVID-19 diagnosis compared with 6 months before COVID-19 diagnosis by largest countries of origin.**

|  | **6 months before COVID-19 diagnosis** | **6 months after COVID-19 diagnosis** | |
| --- | --- | --- | --- |
|  | **OR (95% CI)** | **Unadjusted**  **OR (95% CI)** | **Adjusted**  **OR (95% CI)** |
| Denmark | 1.00 (reference) | 4.14 (4.09 to 4.19) | 4.30 (4.25 to 4.36) |
| Norway | 1.00 (reference) | 2.59 (2.08 to 3.21) | 2.69 (2.13 to 3.39) |
| Sweden | 1.00 (reference) | 5.22 (4.26 to 6.39) | 5.89 (4.70 to 7.37) |
| Afghanistan | 1.00 (reference) | 4.61 (3.95 to 5.37) | 4.67 (3.87 to 5.63) |
| Iraq | 1.00 (reference) | 3.04 (2.73 to 3.38) | 3.68 (3.25 to 4.17) |
| Iran | 1.00 (reference) | 4.52 (3.91 to 5.23) | 5.16 (4.40 to 6.04) |
| Somalia | 1.00 (reference) | 2.74 (2.31 to 3.26) | 3.24 (2.67 to 3.93) |
| Pakistan | 1.00 (reference) | 3.18 (2.84 to 3.57) | 3.38 (2.98 to 3.83) |
| Turkey | 1.00 (reference) | 3.50 (3.25 to 3.77) | 3.92 (3.61 to 4.25) |

Hospital contacts related to any long COVID symptoms included fatigue, headache, dyspnoea (difficulty in breathing), cough, chest pain, depression and/or anxiety as a composite outcome. The adjusted model composed age, sex, civil status, education, family income, and Charlson comorbidity index. OR=odds ratio. CI=confidence interval.
