# Supplementary material for: Risk of long COVID and associated symptoms after acute SARS-COV-2 infection in ethnic minorities: A nationwide register-linked cohort study in Denmark
Source: PLoS Med. 2024 Feb 20;21(2):e1004280. doi: 10.1371/journal.pmed.1004280 (PMC10914299; doi:10.1371/journal.pmed.1004280)
Supplement: S12 Table — Hospital contacts related to any long COVID symptoms included fatigue, headache, dyspnoea (difficulty in breathing), cough, chest pain, depression, and/or anxiety as a composite outcome. The adjusted model composed age, sex, civil status, education, family income, and CCI. CCI, Charlson comorbidity index; CI, confidence interval; OR, odds ratio. (DOCX) [file pmed.1004280.s012.docx]

**S12 Table. Odds ratios of hospital contacts related to any long COVID symptoms by largest countries of origin.**

|  | **6 months before COVID-19 diagnosis** | | | **0 to 4 weeks after COVID-19 diagnosis** | | | **>4 weeks to 6 months after COVID-19 diagnosis** | | |
| --- | --- | --- | --- | --- | --- | --- | --- | --- | --- |
|  | **n (%)** | **Unadjusted**  **OR (95% CI)** | **Adjusted**  **OR (95% CI)** | **n (%)** | **Unadjusted**  **OR (95% CI)** | **Adjusted**  **OR (95% CI)** | **n (%)** | **Unadjusted**  **OR (95% CI)** | **Adjusted**  **OR (95% CI)** |
| **Hospital contacts related to any long COVID symptoms** | | | | | | | | | |
| Denmark  (n=1 952 021) | 25 375 (1.3) | 1.00 (reference) | 1.00 (reference) | 6506 (0.3) | 1.00 (reference) | 1.00 (reference) | 17 516 (0.9) | 1.00 (reference) | 1.00 (reference) |
| Norway  (n=7200) | 83 (1.1) | 0.91 (0.79 to 1.05) | 0.88 (0.75 to 1.03) | 14 (0.2) | 0.74 (0.54 to 1.01) | 0.75 (0.54 to 1.04) | 62 (0.8) | 0.99 (0.83 to 1.17) | 1.04 (0.86 to 1.25) |
| Sweden  (n=7078) | 81 (1.1) | 0.87 (0.74 to 1.01) | 0.81 (0.68 to 0.97) | 23 (0.3) | 0.73 (0.53 to 1.01) | 0.76 (0.54 to 1.07) | 81 (1.1) | 1.57 (1.36 to 1.80) | 1.74 (1.50 to 2.02) |
| Afghanistan (n=9273) | 178 (1.9) | 1.25 (1.11 to 1.40) | 1.04 (0.91 to 1.19) | 52 (0.5) | 1.73 (1.44 to 2.09) | 1.60 (1.28 to 1.99) | 170 (1.8) | 1.91 (1.70 to 2.14) | 1.31 (1.13 to 1.51) |
| Iraq  (n=15 600) | 382 (2.4) | 1.51 (1.40 to 1.62) | 1.19 (1.09 to 1.30) | 118 (0.7) | 1.73 (1.51 to 1.98) | 1.50 (1.29 to 1.75) | 280 (1.7) | 1.71 (1.57 to 1.87) | 1.26 (1.14 to 1.39) |
| Iran  (n=9200) | 210 (2.2) | 1.57 (1.41 to 1.74) | 1.42 (1.27 to 1.59) | 60 (0.6) | 2.37 (2.01 to 2.79) | 2.26 (1.89 to 2.70) | 133 (1.4) | 1.79 (1.58 to 2.01) | 1.59 (1.40 to 1.81) |
| Somalia  (n=9077) | 151 (1.6) | 1.38 (1.22 to 1.55) | 1.10 (0.96 to 1.26) | 47 (0.5) | 1.61 (1.30 to 2.00) | 1.51 (1.20 to 1.90) | 121 (1.3) | 1.68 (1.46 to 1.92) | 1.24 (1.06 to 1.44) |
| Pakistan  (n=12 462) | 278 (2.2) | 1.07 (0.98 to 1.16) | 0.94 (0.86 to 1.04) | 95 (0.7) | 1.43 (1.24 to 1.65) | 1.25 (1.07 to 1.47) | 217 (1.7) | 1.64 (1.51 to 1.79) | 1.37 (1.25 to 1.50) |
| Turkey (n=35 460) | 749 (2.1) | 1.29 (1.23 to 1.37) | 1.07 (1.01 to 1.14) | 284 (0.8) | 2.06 (1.89 to 2.24) | 1.93 (1.75 to 2.12) | 666 (1.8) | 1.73 (1.63 to 1.83) | 1.32 (1.24 to 1.42) |

Hospital contacts related to any long COVID symptoms included fatigue, headache, dyspnoea (difficulty in breathing), cough, chest pain, depression and/or anxiety as a composite outcome. The adjusted model composed age, sex, civil status, education, family income, and Charlson comorbidity index. OR=odds ratio. CI=confidence interval.
