# Supplementary material for: Risk of long COVID and associated symptoms after acute SARS-COV-2 infection in ethnic minorities: A nationwide register-linked cohort study in Denmark
Source: PLoS Med. 2024 Feb 20;21(2):e1004280. doi: 10.1371/journal.pmed.1004280 (PMC10914299; doi:10.1371/journal.pmed.1004280)
Supplement: S13 Table — Northern Europe indicates Northern Europe other than Denmark. (DOCX) [file pmed.1004280.s013.docx]

**S13 Table. Pattern of general practitioner contacts before and during COVID-19 pandemic among individuals diagnosed with COVID-19.**

|  | **January 2018 to December 2018** | **January 2019 to December 2019** | **January 2020 to December 2020** | **January 2021 to December 2021** | **January 2022 to August 2022** |
| --- | --- | --- | --- | --- | --- |
| Denmark (n=1 952 021) | 974 327 (49.9%) | 982 396 (50.3%) | 1 021 263 (52.3%) | 1 011 337 (51.8%) | 738 861 (37.9%) |
| Northern Europe (n=19 842) | 8306 (41.9%) | 8800 (44.3%) | 9784 (49.3%) | 10 246 (51.6%) | 7460 (37.6%) |
| Western Europe (n=37 300) | 13 172 (35.3%) | 14 114 (37.8%) | 16 144 (43.3%) | 17 340 (46.5%) | 12 344 (33.1%) |
| Eastern Europe (n=125 517) | 53 855 (42.9%) | 55 785 (44.4%) | 59 708 (47.6%) | 61 384 (48.9%) | 45 581 (36.3%) |
| Asia (n=62 192) | 28 806 (46.3%) | 29 621 (47.6%) | 31 219 (50.2%) | 31 823 (51.2%) | 23 165 (37.2%) |
| Middle East (n=59 138) | 30 073 (50.9%) | 30 502 (51.6%) | 31 495 (53.3%) | 31 719 (53.6%) | 24 561 (41.5%) |
| North Africa (n=8693) | 4398 (50.6%) | 4469 (51.4%) | 4707 (54.1%) | 4688 (53.9%) | 3510 (40.4%) |
| Subsaharan Africa (n=22 252) | 10 285 (46.2%) | 10 516 (47.3%) | 11 143 (50.1%) | 11 235 (50.5%) | 7870 (35.4%) |

Northern Europe indicates Northern Europe other than Denmark.
