# Supplementary figures and images for: Risk of long COVID and associated symptoms after acute SARS-COV-2 infection in ethnic minorities: A nationwide register-linked cohort study in Denmark
Source: PLoS Med. 2024 Feb 20;21(2):e1004280. doi: 10.1371/journal.pmed.1004280 (PMC10914299; doi:10.1371/journal.pmed.1004280)

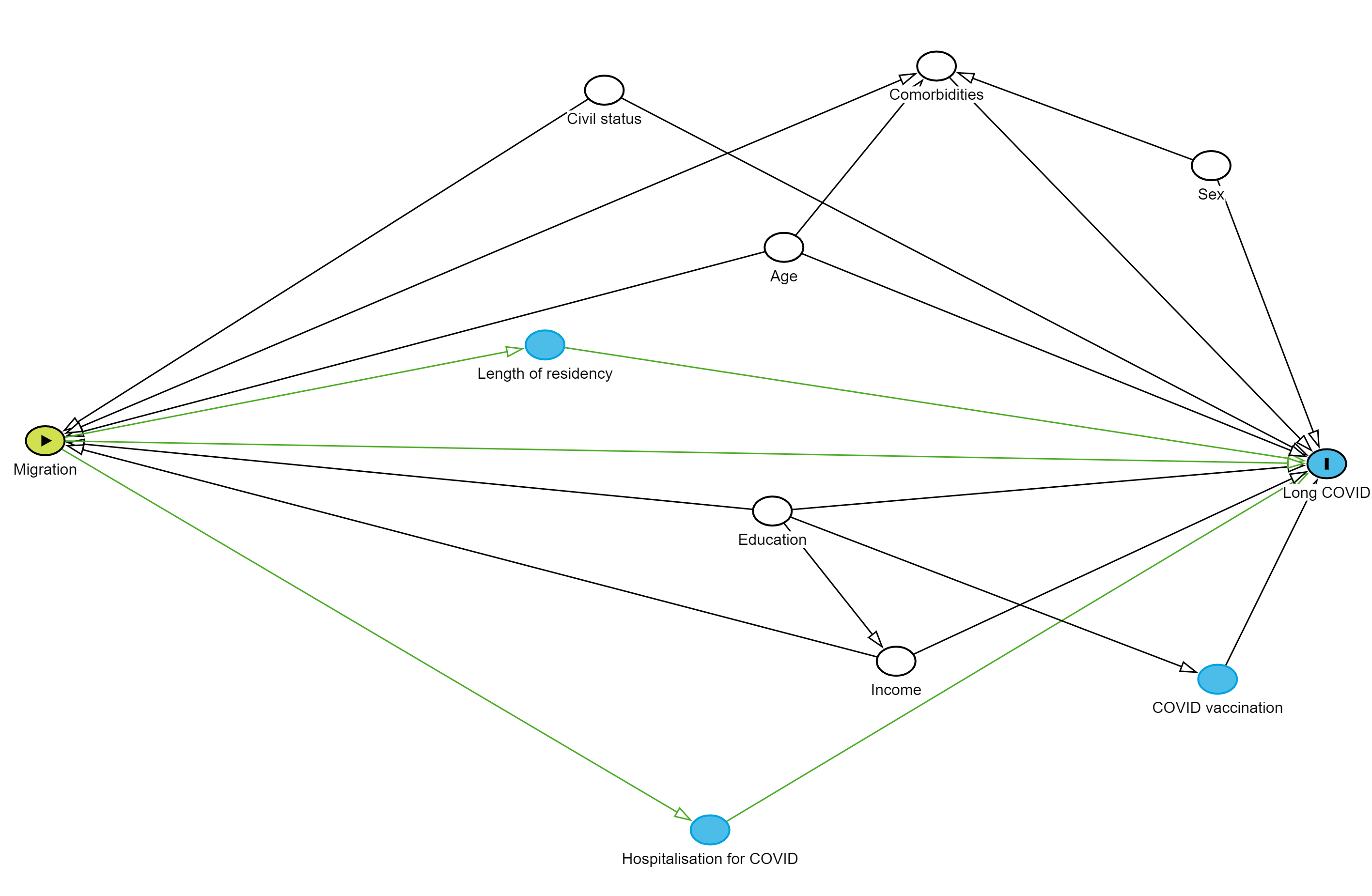

Supplement: S1 Fig — Green lines indicate the pathway of mediators. Black lines indicate the pathway of confounders. Blue circles indicate mediating factors. White circles indicate confounding factors. Age, sex, civil status, comorbidities, education, and income were identified as confounders. (TIFF) [file pmed.1004280.s014.tiff]

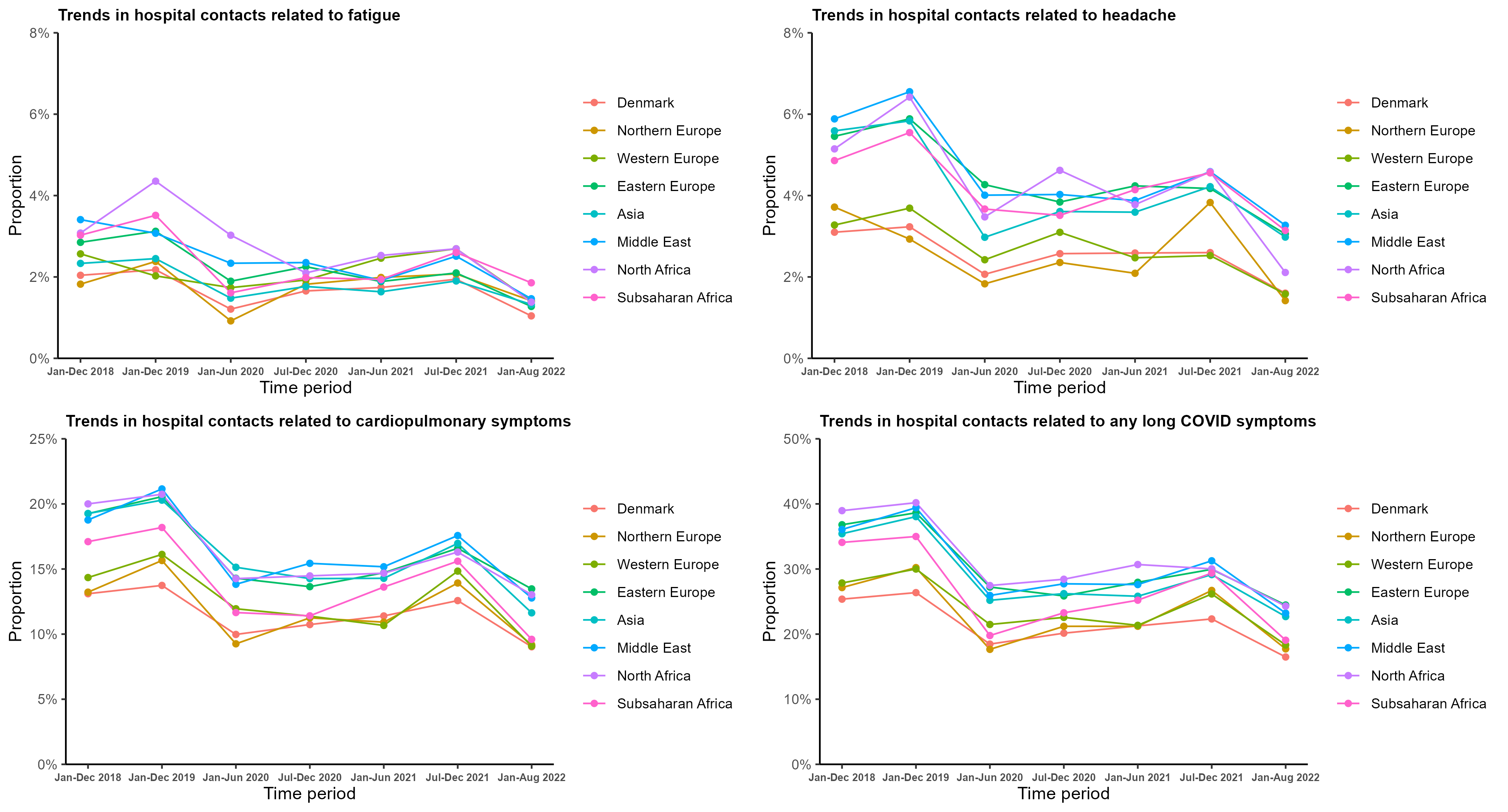

Supplement: S2 Fig — Northern Europe indicates Northern Europe other than Denmark. Hospital contacts related to cardiopulmonary symptoms included dyspnoea (difficulty in breathing), cough, and chest pain as a composite outcome. Hospital contacts related to any long COVID symptoms included fatigue, headache, dyspnoea (difficulty in breathing), cough, chest pain, depression, and/or anxiety as a composite outcome. (TIFF) [file pmed.1004280.s015.tiff]
