# Supplementary material for: Risk of long COVID and associated symptoms after acute SARS-COV-2 infection in ethnic minorities: A nationwide register-linked cohort study in Denmark
Source: PLoS Med. 2024 Feb 20;21(2):e1004280. doi: 10.1371/journal.pmed.1004280 (PMC10914299; doi:10.1371/journal.pmed.1004280)
Supplement: S1 Study Protocol — (DOCX) [file pmed.1004280.s017.docx]

**COVID-19 long-term outcomes/heath consequences among migrants in Denmark: a nationwide register-based study.**

*By George Mkoma and Marie Norredam*

**1. Background**

In April 2020, emerging evidence from the United States and the United Kingdom suggested that migrants and ethnic minorities were particularly hard-hit by corona virus 2019 (COVID-19) illness compared to the majority population (1). Subsequent, findings from the Nordic countries including Denmark and Sweden have also shown higher incidence rates of positive SARS-Cov-2 tests among ethnic minority groups (2). In Denmark, non-Western migrants and descendants accounted for 26% of cases (until 7 September) (3), but only constituted 10% of the total Danish population (Statistics Denmark, 2019). Yet published data further shows that non-Western migrants and descendants with the same age and sex as the majority population had 2.5 times higher risk of being hospitalised with COVID-19 illness. This was especially the case for individuals from Pakistan, Somalia, Morocco and Turkey. At least half of the excess risk was attributed to comorbidity, population density, having a frontline job and household size (4).

Existing knowledge indicates that this overrepresentation of migrants and ethnic minorities among those tested positive for severe acute respiratory syndrome coronavirus 2 (SARS-CoV-2) and/or hospitalised for COVID-19 most likely is caused by several interacting factors such as socioeconomic conditions, high burden of comorbidities, language barriers and sociocultural norms in this population (5–8). The striking overrepresentation of migrants and ethnic minorities in the COVID-19 pandemic are worrying. Yet, until July 12, 2020, globally, less than 0.01% of the all scientific papers (30 599) on COVID-19 included ethnic and racial inequalities probably due to the lack of data collected and shared by public health authorities as well as these data not being collected for research studies (9). In this context, our collaborative study presents a unique possibility of access to large-scale nationwide data as well as hospital cohorts that include information on ethnicity and migration background. Simultaneously, with these ethnic inequalities in COVID-19 we observe a disquieting number of patients with long-term health consequences of COVID-19 leading to the natural conclusion that migrants and ethnic minorities will be disproportionally hard hit by COVID-19 long-term health consequences (long COVID) and that we should not overlook this problem. Long-term health consequences of COVID-19 illness include persistent fatigue, neurological difficulties like depression, headache, difficulty with thinking and concentration as well as and cardiopulmonary symptoms including cough, shortness of breath and chest pain (10-14). However, the literature is scarce.

**2. Objectives**

This project investigates the following research question:

***What is the incidence and risk factors of COVID-19 long-term symptoms and outcomes among migrants and ethnic minorities in Denmark with SARS-CoV-2/COVID-19 illness compared to the majority population?***

Our specific objectives will focus on the following themes:

1. **Substudy A**. We will study overall differences in the incidence of *COVID-19 long-term symptoms and long COVID diagnosis* between migrants and majority population (Danish-born residents). In addition, we will investigate the role of factors such as age, migration status (refugee, family reunified and work), comorbidities, and socioeconomic factors, hospitalisation, and vaccination status in the incidence of long COVID.
2. **Substudy B**. We will compare the risk of utilisation of various medications *post COVID-19* between migrants and Danish-born residents taking into account hospitalisation and/or receiving intensive care, the waves of the pandemic, duration of residence, and age at arrival.
3. **Substudy C**. We will examine the risk of *COVID-19 long-term healthcare utilisation and readmissions* between migrants and Danish-born residents with respect to i) general practitioner contacts, telephone consultations, home visits, preventive consultations, e-mail consultations and out of hour’s services ii) readmissions *after COVID-19 illness*, iii) referral to rehabilitation services, iv) number of contacts for physical exercise and dietary advice. If possible, consequences on labour market and sick leave will be explored.

**3. Methods**

***Data Sources and Study Population***

The project is based on two study populations from Danish Health Data Authority. The first population will include all Danish residents who had at least one diagnosis of SARS-CoV-2/COVID-19 from 1 January 2020 until date of extraction (approximately 1.750.000 patients). The second population includes all individuals who were admitted with SARS-CoV-2/COVID-19 from 1 January 2020 until date of extraction (approximately 35.000 patients). The study populations will be patients who were ≥17 years of age, respectively at the time of COVID-19 test (subpopulation 1) and admission (subpopulation 2).

Both populations will be linked to the following registers, see the variables list: *COVID Database, Danish National Patient Registry (LPR3), Danish National Prescription Registry (DNPR), Danish National Health Service Register, Danish Register of Causes of Death, Intensive care database*.

Populations retrieved from Danish Health Data Authority will be forwarded to Statistics Denmark for further linkages.

***Ethics***

This project was approved by the Danish Data Protection Agency, reference number 514-0670/21-3000 and adheres to the principles of the Declaration of Helsinki. Through Statistics Denmark we will work with data anonymously via remote line access.

***Organisation and publications***

The project will be conducted by George Mkoma, a Postdoc at Department of Public Health, University of Copenhagen together with Principal Investigator Marie Nørredam, who is a Professor in Migration and Health at the Danish Research Centre for Migration, Ethnicity and Health, University of Copenhagen (UCPH) and reservelæge at Department of Infectious Diseases, Hvidovre Hospital. A collaborator, Professor Mikael Rostila from Department of Public Health, Stockholm University, who is an expert in health inequality, social determinants of health, and migration and health research. Further, another collaborator is Professor Charles Agyemang from Amsterdam University Medical Centers, University of Amsterdam, who has an extensive knowledge in migration and health research focusing on non-communicable diseases among ethnic minorities in Europe. Results will be published in international peer-reviewed journals and through seminar.

**4. References**

1. Intensive Care National Audit & Research Centre (ICNARC). ICNARC report on COVID-19 in critical care. United Kingdom, April 8th 2020.
2. Diaz E, Norredam M, Aradhya S, et al. Situational brief: Migration and COVID-19 in

Scandinavian countries, 2020.

1. Statens Serum Institut. COVID-19 i Danmark - Epidemiologisk trend og fokus. Copenhagen, Denmark, 2020. <https://www.ssi.dk/aktuelt/nyheder/2020/status-pa-covid-19-smitte-blandt-etniske-minoriteter-i-danmark>
2. Islamoska S, Holm Petersen J, Benfield T, Norredam M. Socio-economic and Demographic Risk Factors in COVID-19 Hospitalization among Migrants and Ethnic Minorities*. European Journal of Public Health*. 2021:ckab186.
3. Bhala N, Curry G, Martineau AR, Agyemang C, Bhopal R. Sharpening the global focus on ethnicity and race in the time of COVID-19. *Lancet*. 2020;395(10238):1673-1676.
4. Agyemang C, Richters A, Jolani S*,* et al. Ethnic minority status as social determinant for COVID-19 infection, hospitalisation, severity, ICU admission and deaths in the early phase of the pandemic: a meta-analysis. *BMJ Global Health* 2021;6**:**e007433.
5. Andersen GS, Kamper-Jørgenen Z, Carsten B, Norredam M, Bygbjerg IC, Jørgensen ME. Diabetes among migrants in Denmark: incidence, mortality, and prevalence based on a longitudinal register study of the entire population. Diabetes Res Clin Prac 2016;122:9-16.
6. Mkoma GF, Johnsen SP, Iversen HK*,* Andersen G, Norredam M. Incidence of stroke, transient ischaemic attack and determinants of poststroke mortality among immigrants in Denmark, 2004‒2018: a population-based cohort study*. BMJ Open*2021;11**:**e049347.
7. Abuelgasim E, Saw L, Shirke M, Zeinah M, Harky A. COVID-19: Unique public health issues facing Black, Asian and minority ethnic communities. *Current Problems in Cardiology* 2020;45(8): 100621.
8. Huang C, Huang L, Wang Y et al. 6-month consequences of COVID-19 in patients discharged from hospital: a cohort study. *Lancet*. 2021 Jan 8:S0140-6736(20)32656-8. doi: 10.1016/S0140-6736(20)32656-8. Epub ahead of print. PMID: 33428867.
9. Rogers JP, Chesney E, Oliver D et al. Psychiatric and neuropsychiatric presentations associated with severe coronavirus infections: a systematic review and meta-analysis with comparison to the COVID-19 pandemic. *Lancet Psychiatry*. 2020;7(7):611-627.
10. Lambert N. COVID-19 “Long Hauler” Symptoms Survey Report. Accessed on Jan 20th 2021 on: <https://dig.abclocal.go.com/wls/documents/2020/072720-wls-covid-symptom-study-doc.pdf>
11. Carfì A, Bernabei R, Landi F; Gemelli Against COVID-19 Post-Acute Care Study Group. Persistent Symptoms in Patients After Acute COVID-19. *JAMA*. 2020; 324(6): 603–605.
12. Lopez-Leon S, Wegman-Ostrosky T, Perelman C, et al. More than 50 long-term effects of COVID-19: a systematic review and meta-analysis. *Scientific Report*. 2021;11(1):16144.

**Table of list of registers and variables**

| **Register** | **Variables** |
| --- | --- |
| COVID Database | CPR, date of the COVID-19 test, positive test results |
| Danish National Patient Registry | CPR, hospitalisation date, discharge date, treatment in intensive care unit, and use of ventilators, comorbidities, readmission date, rehabilitation date, date and number of physical exercise, date and number of dietary advice.  Myocardial infarction (ICD10, I21−I22), heart failure (ICD10, I50−I50.9), peripheral vascular disease (ICD10, I73), atrial fibrillation (ICD10, I48−I48.9), hypertension (ICD10, I10−I15), cerebrovascular disease/stroke (ICD10, I60−I69), chronic obstructive pulmonary disease (ICD10, J44−J44.9), liver disease (ICD10, K70−K77), diabetes mellitus (ICD10, E10−E14), chronic kidney disease (N18−N18.9), malignant neoplasms (ICD10, C00−C97), dementia (ICD10, F01−F03), and depression symptoms/disorder (ICD10, F32−F32.9), anxiety symptoms/disorders (ICD10, F41−F41.9), bipolar disorder (ICD10, F31−F31.9), schizophrenia (ICD10, F20−F20.9), psychiatric illness (F30-F39, F40-F48) and dyspnea (ICD10, R06−R06.8), chest pain (ICD10, R07−R07.4), cough (ICD10, R05), arrhythmia and bradycardia (ICD10, R00−R00.8), disturbance of smell and taste (ICD10, R43−R43.8), headache and pain (ICD10, R51−R52), general body malaise (ICD10, R53), migraine (ICD10, G43−G43.9), fever (ICD10, R50.9), joint pain (ICD10, M25.5), muscle pain (ICD10, M79.1), nausea and vomiting (ICD10, R11) and dyspepsia (ICD10, K30), COVID diagnosis (ICD 10, U07.1) and long COVID (ICD 10 , U08, U09.9*)* |
| Danish Vaccination Register | CPR and date of vaccination |
| Danish National Health Service Register | CPR, date and number of ordinary consultation to general practitioners, referrals to specialist, telephone consultations, home visits, preventive consultations, e-mail consultations and out of hour’s services. |
| Danish National Prescription Registry | CPR, date of dispensing (expedition date), dosage, ATC medication codes for chest pain (B01A, C01D and all derivatives), cough suppressants (R05DA04, R05DA09R05C), antiarrhythmic (C01B), paracetamol (N02BE01) analgesics and antimigraine (N02A, N02B, N02C, N02CC), NSAIDs (M01A excluding. M01AX), antiemetic and antinauseants (A04A and all derivatives), antipsychotics (NO5A), antidepressants (N06A) and anxiolytics/benzodiazepines (N05BA, N05CF), Platelet inhibitors (B01AC06, B01AC07, B01AC22, B01AC24, B01AC30, N02BA01, B01AC04), bronchodilators (R03AC02-04, R03AC12-13, R03AC18-19, R03AK06-08, R03AK10-11, R03AL01-09, R03CC02, R03BB01, R03BB04- 07), short-acting beta agonists (R03AC02-04, R03AL01-02, R03CC02), inhaled corticosteroids (R03BA, R0AK, R03AL08, R03AL09), anticoagulants (B01AA03, B01AA04, B01AF01- B01AF03, B01AE07), and glucose-lowering drugs (A10) |
| Danish Register of Causes of Death | CPR, date of death and cause of death |
| Statistics Denmark | Demografi, familie og husstandsforhold (BEF), Historiske vandringer (VNDS), Indvandrere og efterkommere (IEPE), Opholdsgrundlag for indvandrede (OPHG), Uddannelser (UDDA), Arbejdsmarkedsklassifikation (AKM), Beskæftigelse for lønmodtagere (BFL), Boligforhold (BOL), Indkomst (IND), Lønoplysninger (IDAP), Familieindkomster (FAIK), Bystørrelse og bopæl (BYSTRB) |

**Variables requested from Statistics Denmark.**

**Registre, der ønskes variable fra**

1. Demografi, familie og husstandsforhold (BEF)
2. Historiske vandringer (VNDS)
3. Indvandrere og efterkommere (IEPE)
4. Opholdsgrundlag for indvandrede (OPHG)
5. Uddannelser (UDDA)
6. Arbejdsmarkedsklassifikation (AKM)
7. Beskæftigelse for lønmodtagere (BFL)
8. Boligforhold (BOL)
9. Indkomst (IND)
10. Lønoplysninger (IDAP)
11. Familieindkomster (FAIK)
12. Bystørrelse og bopæl (BYSTRB)
13. Data over seneste dødsfald (CIV)
14. SGDP – Sygedagpenge – sager
15. SOCP - Sociale pensioner
16. VEUV - Kursister ved voksen- og efteruddannelse
17. RAS - Registerbaserede arbejdsstyrkestatistik

**Populationen**

Projektet baserer sig på 2 populationer fra Sundhedsdatastyrelsen. Den første inkluderer alle Danske borgere, som er blevet diagnosticeret mindst én gang med SARS-CoV-2/COVID-19 fra 1 januar 2020 til udtræksdatoen (ca. 1.750.000 personer). Den anden population er baseret på alle individer, som har været indlagt med SARS-CoV-2/COVID-19 fra 1 januar 2020 til udtræksdatoen (ca. 35.000 patienter). De to studie populationer indeholder kun individer, som er ≥17 når de bliver testet positive (subpopulation 1) eller indlagt (subpopulation 2).

**Udtræks- og variabeloversigt**

Populationen tilføjes følgende registre og variable fra Danmarks Statistik. Såfremt det er muligt, ønskes data så langt frem til seneste opdatering i 2022 som muligt for alle variable.

***Befolkningsregisteret***

| Grundregisternavn  Variabelnavn | BEF  Beskrivelse | År |
| --- | --- | --- |
| PNR | Personnummer | 1.1.2018- seneste opdatering |
| [ADRESSE_ID](https://www.dst.dk/da/Statistik/dokumentation/Times/CPR-oplysninger/ADRESSE-ID) | Bopælsadresse_id | 1.1.2018- seneste opdatering |
| CIVST | Civilstand | 1.1.2018- seneste opdatering |
| [CIV_VFRA](https://dst.dk/da/TilSalg/Forskningsservice/Dokumentation/hoejkvalitetsvariable/Folketal/CIV-VFRA) | Civilstandsdato | 1.1.2018- seneste opdatering |
| [FOEDREG_KODE](https://dst.dk/da/TilSalg/Forskningsservice/Dokumentation/hoejkvalitetsvariable/Folketal/FOEDREG-KODE) | Kode for personens fødselsregistreringssted | 1.1.2018- seneste opdatering |
| [KOEN](https://dst.dk/da/TilSalg/Forskningsservice/Dokumentation/hoejkvalitetsvariable/Folketal/KOEN) | Køn | 1.1.2018- seneste opdatering |
| [FOED_DAG](https://dst.dk/da/TilSalg/Forskningsservice/Dokumentation/hoejkvalitetsvariable/Folketal/FOED-DAG) | Fødselsdato | 1.1.2018- seneste opdatering |
| E_FAELLE_ID | E_fælle | 1.1.2018- seneste opdatering |
| [AEGTE_ID](https://dst.dk/da/TilSalg/Forskningsservice/Dokumentation/hoejkvalitetsvariable/Folketal/AEGTE-ID) | Ægtefælle ID | 1.1.2018- seneste opdatering |
| [FAMILIE_ID](https://dst.dk/da/TilSalg/Forskningsservice/Dokumentation/hoejkvalitetsvariable/Familier/FAMILIE-ID) | Familiens identificerende nummer. | 1.1.2018- seneste opdatering |
| [IE_TYPE](https://dst.dk/da/TilSalg/Forskningsservice/Dokumentation/hoejkvalitetsvariable/Udlaendinge/IE-TYPE) | Indvandrere, efterkommere, personer med dansk oprindelse | 1.1.2018- seneste opdatering |
| [FOERSTE_INDVANDRING](https://www.dst.dk/da/Statistik/dokumentation/Times/Moduldata-for-Befolkning-og-valg/FOERSTE-INDVANDRING) | Første indvandringsdato | 1.1.2018- seneste opdatering |
| [SENESTE_INDVANDRING](https://www.dst.dk/da/Statistik/dokumentation/Times/Moduldata-for-Befolkning-og-valg/SENESTE-INDVANDRING) | Seneste indvandringsdato | 1.1.2018- seneste opdatering |
| [HUSTYPE](https://dst.dk/da/TilSalg/Forskningsservice/Dokumentation/hoejkvalitetsvariable/Husstande/HUSTYPE) | Husstandstype | 1.1.2018- seneste opdatering |
| [FAMILIE_TYPE](https://dst.dk/da/TilSalg/Forskningsservice/Dokumentation/hoejkvalitetsvariable/Familier/FAMILIE-TYPE) | Familietype | 1.1.2018- seneste opdatering |
| [STATSB](https://www.dst.dk/da/Statistik/dokumentation/Times/moduldata-for-befolkning-og-valg/statsb) | Statsborgerskab | 1.1.2018- seneste opdatering |
| [PLADS](https://dst.dk/da/TilSalg/Forskningsservice/Dokumentation/hoejkvalitetsvariable/Familier/PLADS) | Familiestatus | 1.1.2018- seneste opdatering |
| [OPHOLDMD_DK](https://www.dst.dk/da/Statistik/dokumentation/Times/Moduldata-for-Befolkning-og-valg/OPHOLDMD-DK) | Opholdstid i måneder | 1.1.2018- seneste opdatering |
| [OPR_LAND](https://dst.dk/da/TilSalg/Forskningsservice/Dokumentation/hoejkvalitetsvariable/Udlaendinge/OPR-LAND) | Oprindelsesland | 1.1.2018- seneste opdatering |
| [VAN_VTIL](https://dst.dk/da/TilSalg/Forskningsservice/Dokumentation/hoejkvalitetsvariable/Indvandringer-til-Danmark/VAN-VTIL) | Indvandringsdato | 1.1.2018- seneste opdatering |
| [KOM](https://dst.dk/da/TilSalg/Forskningsservice/Dokumentation/hoejkvalitetsvariable/Folketal/KOM) | Ingen label | 1.1.2018- seneste opdatering |
| [ANTBOERNF](https://dst.dk/da/TilSalg/Forskningsservice/Dokumentation/hoejkvalitetsvariable/Familier/ANTBOERNF) | Antal børn i familien | 1.1.2018- seneste opdatering |
| [ANTBOERNH](https://dst.dk/da/TilSalg/Forskningsservice/Dokumentation/hoejkvalitetsvariable/Husstande/ANTBOERNH) | Antal børn i husstanden | 1.1.2018- seneste opdatering |
| [ANTEFAM](https://www.dst.dk/da/Statistik/dokumentation/Times/Moduldata-for-Befolkning-og-valg/ANTEFAM) | Antal E-familier i husstanden | 1.1.2018- seneste opdatering |
| [ANTPERSF](https://www.dst.dk/da/Statistik/dokumentation/Times/Moduldata-for-Befolkning-og-valg/ANTPERSF) | Antal personer i familien | 1.1.2018- seneste opdatering |
| [ANTPERSH](https://dst.dk/da/TilSalg/Forskningsservice/Dokumentation/hoejkvalitetsvariable/Husstande/ANTPERSH) | Antal personer i husstanden | 1.1.2018- seneste opdatering |
| [VERSION](https://www.dst.dk/da/Statistik/dokumentation/Times/Moduldata-faelles-variable/VERSION) | Moduldata version | 1.1.2018- seneste opdatering |

***Historiske vandringer***

| Grundregisternavn: | VNDS |  |
| --- | --- | --- |
| Variabelnavn | **Beskrivelse** | År |
| PNR | Personnummer | 1.1.2018- seneste opdatering |
| HAEND_DATO | Hændelsesdato | 1.1.2018- seneste opdatering |
| INDUD_KODE | Ingen label | 1.1.2018- seneste opdatering |
| INDUD_LAND | Ingen label | 1.1.2018- seneste opdatering |

***Indvandrere og efterkommere***

| Grundregisternavn  Variabelnavn | IEPE  Beskrivelse | År |
| --- | --- | --- |
| PNR | Personnummer | 1.1.2018- seneste opdatering |
| GENERATION | Kategorisering af 2. og 3. generationsindvandrere (efter DS. definition) | 1.1.2018- seneste opdatering |
| [IE_TYPE](https://dst.dk/da/TilSalg/Forskningsservice/Dokumentation/hoejkvalitetsvariable/Udlaendinge/IE-TYPE) | Indvandrere, efterkommere , personer med dansk oprindelse | 1.1.2018- seneste opdatering |
| [OPR_LAND](https://dst.dk/da/TilSalg/Forskningsservice/Dokumentation/hoejkvalitetsvariable/Udlaendinge/OPR-LAND) | Oprindelsesland | 1.1.2018- seneste opdatering |
| VERDEL1 | VERDEL1 | 1.1.2018- seneste opdatering |
| VEST_EJ | VEST_EJ | 1.1.2018- seneste opdatering |

***Opholdsgrundlag for indvandrede***

| Grundregisternavn | OPHG |  |
| --- | --- | --- |
| Variabelnavn | **Beskrivelse** | År |
| PNR | Personnummer | 1.1.2018- seneste opdatering |
| FORKLAR | Opholdstilladelsens forklaring | 1.1.2018- seneste opdatering |
| GRUNDLAG | Opholdstilladelsens grundlag | 1.1.2018- seneste opdatering |
| IMPUTERET | Imputeret opholdstilladelse | 1.1.2018- seneste opdatering |
| KATEGORI | Opholdstilladelsens kategori | 1.1.2018- seneste opdatering |
| REFERENCETID | Referencetid | 1.1.2018- seneste opdatering |
| TILLADELSESDATO | Dato for opholdstilladelse | 1.1.2018- seneste opdatering |
| VERSION | Version | 1.1.2018- seneste opdatering |

***Data over seneste dødsfald - CIV registret***

| Grundregisternavn | BEF_CIVILSTAND |  |
| --- | --- | --- |
| Variabelnavn | **Beskrivelse** | År |
| PNR | Personnummer | 1.1.2018- seneste opdatering |
| CIVST | Civilstand og død | 1.1.2018- seneste opdatering |
| CIV_VFRA | Civilstandsdato og dødsdato | 1.1.2018- seneste opdatering |

***Uddannelser***

| Grundregisternavn  Variabelnavn | UDDA  Beskrivelse | | År |
| --- | --- | --- | --- |
| PNR | Personnummer | 1.1.2018- seneste opdatering | |
| [HFAUDD](https://dst.dk/da/TilSalg/Forskningsservice/Dokumentation/hoejkvalitetsvariable/Hoejst-fuldfoerte-uddannelse/HFAUDD) | Højeste fuldførte uddannelse | 1.1.2018- seneste opdatering | |
| [HF_KILDE](https://dst.dk/da/TilSalg/Forskningsservice/Dokumentation/hoejkvalitetsvariable/Hoejst-fuldfoerte-uddannelse/HF-KILDE) | Kilde til uddannelsesoplysningen | 1.1.2018- seneste opdatering | |
| [ERHAUDD](https://www.dst.dk/da/Statistik/dokumentation/Times/Uddannelsesdata/Befolkningens-Uddannelse/ERHAUDD) | Højst fuldførte erhvervskompetencegivende uddannelse | 1.1.2018- seneste opdatering | |
| [UDD](https://dst.dk/da/TilSalg/Forskningsservice/Dokumentation/hoejkvalitetsvariable/Hoejst-fuldfoerte-uddannelse/UDD) | Uddannelseskode | 1.1.2018- seneste opdatering | |
| HF_VFRA | Tidspunkt for opnået højst fuldførte uddannelse | 1.1.2018- seneste opdatering | |

***Arbejdsklassifikationsmodulet***

| Grundregisternavn | AKM | |  |
| --- | --- | --- | --- |
| Variabelnavn | **Beskrivelse** | | År |
| PNR | Personnummer | 1.1.2018- seneste opdatering | |
| FAMILIE_ID | Familiens identificerende nummer | 1.1.2018- seneste opdatering | |
| DISCO08_ALLE_INDK | Fagklassifikation for beskæftigelsesforhold, fra 2010 (AKM) | 1.1.2018- seneste opdatering | |
| DISCO08_ALLE_INDK_13 | Fagkode for væsentligste beskæftigelse i året- fra 2010 (AKM) | 1.1.2018- seneste opdatering | |

***Beskæftigelse for lønmodtagere***

| Grundregisternavn | BFL |  |
| --- | --- | --- |
| Variabelnavn | **Beskrivelse** | År |
| PNR | Personnummer | 1.1.2018- seneste opdatering |
| [AJO_BRANCHE07](https://www.dst.dk/da/Statistik/dokumentation/Times/beskaeftigelse-for-loenmodtagere/ajo-branche07) | DB07 branche knyttet til jobbets arbejdssted | 1.1.2018- seneste opdatering |
| AJO_BREDT_LOENBELOEB | Bredt lønbeløb er en summering af al lønindkomst i eIR dvs. inkl. ATP-bidrag og personalegoder. | 1.1.2018- seneste opdatering |
| AJO_FULDTID_BESKAEFTIGET | Fuldtidsbeskæftigelse | 1.1.2018- seneste opdatering |
| AJO_FUNKTIONSKODE | Funktionskode til sektorplacering af arbejdsstedet | 1.1.2018- seneste opdatering |
| AJO_INDBERETTEDE_LOENTIMER | Løntimer på prodjob | 1.1.2018- seneste opdatering |
| AJO_INDKOMST_ART_KODE | Indkomstartkode på prodjob | 1.1.2018- seneste opdatering |
| AJO_INDKOMST_TYPE_KODE | Indkomsttypekode på prodjob | 1.1.2018- seneste opdatering |
| AJO_JOB_SLUT_DATO | Slutdato for det komprimerede job. | 1.1.2018- seneste opdatering |
| AJO_JOB_START_DATO | Startdato for det komprimerede job. | 1.1.2018- seneste opdatering |
| AJO_LOENTIMER | Løntimer på komprimerede job. | 1.1.2018- seneste opdatering |
| AJO_LOENTIME_KODE | Kode for, om løntimer er indberettet eller imputeret | 1.1.2018- seneste opdatering |
| AJO_SMALT_LOENBELOEB | Smalt lønbeløb, som omfatter lønindkomst uden ATP-bidrag og personalegoder | 1.1.2018- seneste opdatering |
| SEKTORKODE | Sektorkode på arbejdssted | 1.1.2018- seneste opdatering |
| [VMO_DISCO_KODE](https://www.dst.dk/da/Statistik/dokumentation/Times/eindkomstregister/vmo-disco-kode) | DISCO kode | 1.1.2018- seneste opdatering |
| [VMO_DISCO_MATCHPRIO_KODE](https://www.dst.dk/da/Statistik/dokumentation/Times/eindkomstregister/vmo-disco-matchprio-kode) | DISCO match prioriterings kode | 1.1.2018- seneste opdatering |
| VMO_DISCO_SLUT_DATO | Slutdato for DISCO kode | 1.1.2018- seneste opdatering |
| VMO_DISCO_START_DATO | Startdato for DISCO kode på lønindberetning | 1.1.2018- seneste opdatering |

***Boligtællingen***

| Grundregisternavn | BOL | |  |
| --- | --- | --- | --- |
| Variabelnavn | **Beskrivelse** | | År |
|  |  | |  |
| BOPIKOM | Bopæl i kommunen | 1.1.2018- seneste opdatering | |
| KOM | INGEN LABEL | 1.1.2018- seneste opdatering | |
| ANTAL_VAERELSER | Antal værelser i bolig- eller erhvervsenheden 314 | 1.1.2018- seneste opdatering | |
| INSTALLATIONFH | INSTALLATIONFH | 1.1.2018- seneste opdatering | |
| [INSTALLATIONSFORHOLD](https://www.dst.dk/da/Statistik/dokumentation/Times/Bolig---Ejendoms-databasen--BED-/INSTALLATIONSFORHOLD) | Installationsforhold for bolig-/erhvervsenhed | 1.1.2018- seneste opdatering | |
| BOLIGAREAL | Samlet boligareal 217 | 1.1.2018- seneste opdatering | |
| EJERFORHOLD | Ejerforhold for ejendommen - felt F102 iflg. BBR-instruks | 1.1.2018- seneste opdatering | |
| ANTAL_PERSONER | Ingen label | 1.1.2018- seneste opdatering | |
| ANTAL_BOERN | Ingen label | 1.1.2018- seneste opdatering | |
| HUSSTANDE_PER_BOLIG | Ingen label | 1.1.2018- seneste opdatering | |
| BOLIGHUSTYPE | Ingen label | 1.1.2018- seneste opdatering | |
| BYSTOERRELSESKODE | Bystørrelseskode | 1.1.2018- seneste opdatering | |
| UDLEJNINGSFORHOLD | Udlejningsforhold - F261/F322/F392 | 1.1.2018- seneste opdatering | |
| UDLEJNINGFH | Ingen label | 1.1.2018- seneste opdatering | |
| DST_BOLIGMATCH | Ingen label | 1.1.2018- seneste opdatering | |

***Indkomst***

| Grundregisternavn | IND | |  |
| --- | --- | --- | --- |
| Variabelnavn | **Beskrivelse** | | År |
| PNR | Personnummer | 1.1.2015- seneste opdatering | |
| [PERINDKIALT](https://dst.dk/da/TilSalg/Forskningsservice/Dokumentation/hoejkvalitetsvariable/Personindkomster/PERINDKIALT) | Personindkomst i alt ekskl. beregnet lejeværdi af egen bolig og før fradrag af renteudgifter | 1.1.2015- seneste opdatering | |
| [PERINDKIALT_13](https://dst.dk/da/TilSalg/Forskningsservice/Dokumentation/hoejkvalitetsvariable/Personindkomster/PERINDKIALT-13) | Personindkomst i alt ekskl. beregnet lejeværdi af egen bolig og før fradrag af renteudgifter | 1.1.2015- seneste opdatering | |
| [QSPLINDK](https://dst.dk/da/TilSalg/Forskningsservice/Dokumentation/hoejkvalitetsvariable/Personindkomster/QSPLINDK) | Skattepligtig indkomst | 1.1.2015- seneste opdatering | |
| [UDD](https://dst.dk/da/TilSalg/Forskningsservice/Dokumentation/hoejkvalitetsvariable/Hoejst-fuldfoerte-uddannelse/UDD) | Uddannelseskode | 1.1.2015- seneste opdatering | |
| [BESKST](https://www.dst.dk/da/Statistik/dokumentation/Times/Personindkomst/BESKST) | Beskæftigelsesstatus 1980 til 2001 (Indkomst/ AKM) | 1.1.2015- seneste opdatering | |
| BESKST02 | Beskæftigelsesstatus fra 2002 (Indkomst/AKM) | 1.1.2015- seneste opdatering | |
| [BESKST13](https://www.dst.dk/da/Statistik/dokumentation/Times/Personindkomst/BESKST13) | Kode for personens væsentligste indkomst-kilde (INDKOMST/AKM) | 1.1.2015- seneste opdatering | |
| [ERHVERVSINDK_13](https://www.dst.dk/da/Statistik/dokumentation/Times/personindkomst/erhvervsindk-13) | Erhvervsindkomst, løn og nettooverskud af selvstændig virksomhed inkl. visse honorarer | 1.1.2015- seneste opdatering | |
| [ADAGP](https://www.dst.dk/da/Statistik/dokumentation/Times/personindkomst/adagp) | Syge- og barselsdagpenge udbetalt af kommunerne ekskl. udbetaling til selvstændige | 1.1.2015- seneste opdatering | |
| [ADAGPAGN](https://www.dst.dk/da/Statistik/dokumentation/Times/personindkomst/adagpagn) | Arbejdsgiverbetalt sygedagpenge | 1.1.2015- seneste opdatering | |
| [AEKVIVADISP_13](https://www.dst.dk/da/Statistik/dokumentation/Times/personindkomst/aekvivadisp-13) | Ækvivaleret disponibel indkomst | 1.1.2015- seneste opdatering | |
| [QBISTYD](https://www.dst.dk/da/Statistik/dokumentation/Times/personindkomst/qbistyd) | Kontanthjælps-, aktiverings-, revaliderings, integrations-, og ledighedsydelse | 1.1.2015- seneste opdatering | |
| [QBISTYD2](https://www.dst.dk/da/Statistik/dokumentation/Times/personindkomst/qbistyd2) | Skattepligtig kontanthjælps-, aktiverings- og revalideringsydelser | 1.1.2015- seneste opdatering | |
| [OFF_OVERFORSEL_13](https://www.dst.dk/da/Statistik/dokumentation/Times/personindkomst/off-overforsel-13) | Offentlige overførsler | 1.1.2015- seneste opdatering | |
| [OVRIG_KONTANTHJALP_13](https://www.dst.dk/da/Statistik/dokumentation/Times/personindkomst/ovrig-kontanthjalp-13) | Aktiverings, ledighed- og revalideringsydelse mv. | 1.1.2015- seneste opdatering | |
| [OVRIG_OVERFORSEL_13](https://www.dst.dk/da/Statistik/dokumentation/Times/personindkomst/ovrig-overforsel-13) | Andre overførsler. SU, boligstøtte, grøn check og børnetilskud. | 1.1.2015- seneste opdatering | |
| [DISPON_13](https://www.dst.dk/da/Statistik/dokumentation/Times/personindkomst/dispon-13) | Indkomst efter skat og renter tillagt beregnet lejeværdi af egen bolig. | 1.1.2015- seneste opdatering | |

***Lønoplysninger***

| Grundregisternavn | IDAP | |  |
| --- | --- | --- | --- |
| Variabelnavn | **Beskrivelse** | | År |
| PNR | Personnummer | 1.1.2015- seneste opdatering | |
| PSTILL | Primær arbejdsstilling | 1.1.2015- seneste opdatering | |
| SSTILL | Sekundær arbejdsstilling | 1.1.2015- seneste opdatering | |

***Familieindkomster***

| Grundregisternavn | FAIK | |  |
| --- | --- | --- | --- |
| Variabelnavn | **Beskrivelse** | | År |
| FAMILIE_ID | Familiens identificerende nummer. | 1.1.2015- seneste opdatering | |
| FAMSOCIOGRUP_13 | Familiens socioøkonomiske gruppe Def13 | 1.1.2015- seneste opdatering | |
| FAMINDKOMSTIALT_13 | Indkomst i alt, før skatter mv. | 1.1.2015- seneste opdatering | |
| FAMAEKVIVADISP_13 | Ækvivaleret disponibel indkomst for familien | 1.1.2015- seneste opdatering | |
| FAMDISPONIBEL_13 | Disponibel indkomst | 1.1.2015- seneste opdatering | |
| FAMOFF_OVERFORSEL_13 | Familiens offentlige overførsler | 1.1.2015- seneste opdatering | |
| FAMBOLIGFORM | Familiens boligform | 1.1.2015- seneste opdatering | |
| FAMBOLIGSTOETTE | Boligstøtte udbetalt til familien | 1.1.2015- seneste opdatering | |
| FAMBOLIGTYPE | Familiens boligtype | 1.1.2015- seneste opdatering | |

***Bystørrelser bopæle***

| Grundregisternavn: | BYSTRB |  |
| --- | --- | --- |
| Variabelnavn | Beskrivelse | År |
|  |  |  |
| BEBOELSE | Beboelse | 1.1.2018- seneste opdatering |
| DSTKODE | Danmarks Statistik's løbenr. for et byområde | 1.1.2018- seneste opdatering |
| DSTNAVN | DSt's stednavnsangivelse for et byområde (bebyggelse=/> 200 indbyggere) | 1.1.2018- seneste opdatering |
| FOLKETAL | Antal personer boende på adressen | 1.1.2018- seneste opdatering |
| HUSSTANDE | Antal husstande boende på adressen | 1.1.2018- seneste opdatering |
| KOM | Kommune | 1.1.2018- seneste opdatering |
| KOORDTYPE | Metode til hvordan koordinaterne er fundet | 1.1.2018- seneste opdatering |
| OPGIKOM | Opgangsadresse i kommunen | 1.1.2018- seneste opdatering |
| POSTNR | Postnummer | 1.1.2018- seneste opdatering |
| SOGNENR | Sognekode | 1.1.2018- seneste opdatering |
| STORHED | Bystørrelse | 1.1.2018- seneste opdatering |

***SGDP – Sygedagpenge - sager***

| Grundregisternavn | SGDP |  |
| --- | --- | --- |
| Variabelnavn | **Beskrivelse** | År |
| PNR | Personnummer | 1.1.2015-2022 |
| ANTDAGE | Antal dagpengedage i året | 1.1.2018- seneste opdatering |
| ARBGHP | Udbetalt beløb til arbejdsgivere | 1.1.2018- seneste opdatering |
| BERDAGE | Antal dagpengedage i hele sagens forløb | 1.1.2018- seneste opdatering |
| FOERBER | Første dagpengedag | 1.1.2018- seneste opdatering |
| FOERFRAV | Første fraværsdag | 1.1.2018- seneste opdatering |
| FRAVDAGE | Antal fraværsdage | 1.1.2018- seneste opdatering |
| SIDBER | Sidste dagpengedag | 1.1.2018- seneste opdatering |
| SIKRHP | Udbetalt beløb til den sikrede | 1.1.2018- seneste opdatering |

***SOCP – Social pensioner***

| Grundregisternavn | SOCP – Social pensioner |  |
| --- | --- | --- |
| Variabelnavn | **Beskrivelse** | År |
| PNR | Personnummer | 1.1.2015-2022 |
| SLUTPENKODE | Pensionskode 31. december året før. | 1.1.2018- seneste opdatering |
| STARTPENKODE | Start pensionskode | 1.1.2018- seneste opdatering |
| AAR_IALT_BELOEB | Årligt pensionsbeløb i alt, kroner | 1.1.2018- seneste opdatering |
| SOP_STARTDATO | Start dato for pension | 1.1.2018- seneste opdatering |
| FOERTID_BELOEB | Førtidsbeløb for januar, kroner | 1.1.2018- seneste opdatering |

***RAS -  Registerbaserede arbejdsstyrkestatistik***

| Grundregisternavn | VEUV | |  |
| --- | --- | --- | --- |
| Variabelnavn | **Beskrivelse** | | År |
| PNR | Personnummer | 1.1.2015- 2022 | |
| SOC_STATUS_KODE | Socioøkonomisk status | 1.1.2015- seneste opdatering | |
| ARB_BEL_KOM_KODE | Arbejdsstedskommune | 1.1.2015- seneste opdatering | |
| HELTID_32_KODE | Heltid eller deltidsbeskæftiget | 1.1.2015- seneste opdatering | |
| PRIMAER_STATUS_KODE | Kode for primær tilknytning til arbejdsmarkedet | 1.1.2015- seneste opdatering | |

***VEUV -  Kursister ved voksen- og efteruddannelse***

| Grundregisternavn | VEUV |  |
| --- | --- | --- |
| Variabelnavn | **Beskrivelse** | År |
| PNR | Personnummer | 1.1.2015-2019 |
| INSTNR | Institutionsnummer | 1.1.2018-2022 |
| UDD | Uddannelseskode | 1.1.2018-2022 |
| OMR_KODE | OMR_KODE | 1.1.2018-2022 |
| KURSIST_VFRA | Starttidspunkt | 1.1.2018-2022 |
| KURSIST_VTIL | Sluttidspunkt | 1.1.2018-2022 |
| AFSLUTN | Kursets afslutningsmåde | 1.1.2018-2022 |
