# Supplementary material for: Risk of long COVID and associated symptoms after acute SARS-COV-2 infection in ethnic minorities: A nationwide register-linked cohort study in Denmark
Source: PLoS Med. 2024 Feb 20;21(2):e1004280. doi: 10.1371/journal.pmed.1004280 (PMC10914299; doi:10.1371/journal.pmed.1004280)
Supplement: Attachment — Submitted filename: Response to PLOS Medicine_final.docx [file pmed.1004280.s019.docx]

**Response to Comments on Manuscript ID:** PMEDICINE-D-23-02370R1, entitled "Risk of long COVID and associated symptoms after acute SARS-COV-2 infection in ethnic minorities: a Danish nationwide cohort study" by Mkoma GF et al.

Alexandra Schaefer, PhD
Associate Editor
PLOS Medicine

Thank you for giving us the opportunity to submit a revised draft of our manuscript for consideration for publication in *PLOS Medicine*. We appreciate the time and effort that editors and reviewers have dedicated to providing valuable feedback on our manuscript. Below we address each comment point-by-point and provide the exact page number and line number where each revision was made, referencing the marked version of the revised manuscript.

**Requests from the editors:**

General comments:

1. Please include page numbers and line numbers in the manuscript file. Use continuous line numbers (do not restart the numbering on each page).

**Response: We have done as requested.**

2. Please cite your Supporting Information as outlined in PLOS Medicine guideline:

**Response: We have cited the supporting information in the manuscript file as per guideline.**

3. Please remove the Ethics statement, the Financial Disclosure statement, Data availability statement and Competing Interest statement from the main body of the manuscript. These details should only be provided in the corresponding section in the manuscript submission form.

**Response: We have removed the Ethics statement, the Financial Disclosure statement, Data availability statement and Competing Interest statement from the main manuscript.**

4. When you refer to “Northern Europe”, please ensure that you mean “Northern Europe other than Denmark”.

**Response: We have described in the methods section in page 7 line 159 that Northern Europe does not include Denmark. In addition, we have added a legend text underneath every table and figure where applicable to indicate this.**

**Academic editor comments:**

1. Abstract
   *Should read “less is known about long COVID in these populations”

**Response: We have corrected the statement see page 2 line 32.**

*First line under Methods and Findings is not a complete sentence

**Response: We have rephrased the statement. Now the statement reads “We used nationwide register-based cohort data on individuals diagnosed with COVID-19 aged ≥18 years (n=2 286 955) between January 2020 and August 2022 in Denmark” see page 2 line 35–37.**

2. Introduction

*The introduction highlights several studies that have identified disparities across multiple studies but would benefit from clear discussion also highlighting the systemic inequities in access and treatment that have likely led to these disparities in outcomes. In this discussion it is important to recognize that even in settings like Denmark with a robust public health infrastructure and free testing and vaccination, systemic inequities still likely exist that lead to different accesses to trusted information, knowledge about resources, time away from work, etc (could see Saarinen et al. in PLOS Medicine for an example). Any of these that could lead to differences in healthcare utilization that impact outcomes.

**Response: Thanks for this very useful suggestion. We have cited and included the discussion on both individual-related factors (such as socioeconomic status, cultural beliefs, health insurance coverage, and language proficiency) and structural-related factors (such as healthcare providers’ stereotypical views and cultural insensitivity, health policy, geographical location of healthcare facility, and availability of professional medical interpreters) as potential factors that may have contributed to the likelihood of long COVID diagnosis among ethnic minorities, using reflections from Andersen’s and Levesque’s conceptual frameworks of healthcare assess. In addition, we have discussed that although all registered residents in Denmark have free access to healthcare services, disparities have previously been found (see page 4 line 91–107).**

3. Methods

*Is there data on disparities in testing and vaccination uptake? Just because it is free for everyone doesn’t mean access is the same as many other factors going into access (see Levesque et al. for a framework on components of access to health care).

**Response: We did not have data on those who had negative COVID-19 test as this would have allowed us to combine with the presented positive COVID-19 test and subsequently examine if there were disparities in access to testing for COVID-19 in relation to the total number of each ethnic group in the general population. However, in Denmark, testing for COVID-19 infection by PCR was universally implemented since May 2020 and accessing to many public settings required a negative COVID-19 test during the time of pandemic until when COVID-19 was no longer considered a critical threat to society by the Danish government. Therefore, we believe that large number of people had taken multiple number of COVID-19 tests; and thus, ethnic disparities on access to testing were more likely to be minor. Furthermore, we agree with the academic editor that free access to testing is not the same as realized access (actual testing for COVID-19 infection) which may be influenced by both individual- and structural-related factors in accordance with Andersen as well as Levesque models.
Data on vaccination uptake including first, second, and third dose of COVID-19 vaccine by ethnic group are already included in the manuscript, and we found that most ethnic minorities, especially North Africans, Middle Eastern, Subsaharan Africans, and Eastern Europeans had significantly lower uptake of COVID-19 vaccine than native Danes (see in results section page 11–12 line 275–277 and also in Table 1).**

*I am not sure you can say that primary outcome definition is “complications persisting beyond the acute COVID-19 infection that cannot be explained by alternative diagnosis”. This would likely need an adjudication process and detailed review of health records (or at least documentation of a standardized set of tests excluding other reasons). This is conceptually what the authors are trying to capture, but a more appropriate definition is documented ICD-10 codes for long COVID and ICD-10 codes for related symptomatology.

**Response: We have provided more clarification regarding the definition used in categorizing long COVID cases in the Danish setting, which is in accordance with the WHO ICD-10 code; see page 7 line 174–177.**
*Would mediation analyses examining impact of hospitalization and/or vaccination be possible?

**Response: We acknowledge that mediation analyses could have disentangled better the relationship between these mediators (hospitalisation and/or vaccination status) and the risk of long COVID. However, in our main analysis, it is already evident that being hospitalised for COVID-19 is significantly associated with increased risk of long COVID both in ethnic minorities and native Danes (see Table 2). Furthermore, we provided additional analyses investigating the impact of different number of doses of COVID-19 vaccine (see S7 Table). Thus, we felt keeping the same analytical approach would be easier for most readers to follow.**

4. Results

*I wonder if there is the possibility for ascertainment bias particularly related to hospital contacts. If hypotheses about inequities in access are true, ascertainment of these symptoms may have been lower among ethnic minorities compared to native Danes. If ethnic minorities then increased their health care utilization after a COVID-19 diagnose, this would then appear to be an increase in these symptoms when it is really driven by differences in health care seeking behavior. Are there differences in baseline healthcare utilization (not necessarily symptoms but number of primary care visits or other vaccine uptake, etc.)? This is an issue that plagues most research on long COVID, and it is challenging to feel confident in findings in baseline.

**Response: We have included data on number of primary care visits (i.e., general practitioner contacts) before and during COVID-19 pandemic to illustrate the pattern of healthcare utilization among various ethnic groups (see S1 Appendix). The findings show that compared with native Danes, North Africans and Middle Eastern were more likely to contact general practitioner both before and during COVID pandemic. This is to a certain extent in line with the previous work in Denmark showing that most ethnic minorities originating from non-Western countries have increased contacts to emergency room, general practitioners, and specialists (see Nielsen SS et al 2012 Scand J Public Health). Furthermore, we have provided additional analyses on pattern of hospital contacts related to long COVID symptoms before and during COVID pandemic (see S1 Appendix). Again, this analysis shows that most ethnic minorities were more likely than native Danes to report fatigue, headache, and cardiopulmonary symptoms even prior to the pandemic. Hence, we have included the discussion about ethnic minorities’ pattern of healthcare utilisation as one of the contributing factors to their increased risk of long COVID diagnosis (see page 16 line 389–399).**

*Figure 4 seems to somewhat suggest that patterns of disparities existed prior to COVID, but they prevalence of symptoms increased by 3 times. For example, the shape of the differences across ethnic groups remains the same. This would somewhat go against the conclusions, and I think highlights some risk in overinterpreting the findings and occasional statistically significant values.

**Response: Since figure 4 may bring the risk of overinterpretation, we have replaced the reported adjusted proportions with adjusted odds ratios from Table S4 (which is now S10 Table). Additionally, we have provided baseline data on patterns of healthcare utilisation (hospital contacts and general practitioner contacts) by ethnic groups which give a better understanding of healthcare utilisation even before COVID pandemic (see S1 Appendix). The findings show that most ethnic minorities were more likely than native Danes to report fatigue, headache, and cardiopulmonary symptoms even prior to COVID pandemic. In addition, some ethnic minorities (i.e., North Africans and Middle Eastern) were more likely than native Danes to contact general practitioner even before COVID pandemic. Hence, these findings are line with our conclusions.**

*What were differences in symptomology between ethnic minorities when only restricted to pre-COVID period? I think differences pre-COVID would again suggest other underlying etiologies for disparities (e.g., systemic inequities).

**Response: Figures below illustrate both pre-COVID period and during COVID period for each symptom (attached in S1 Appendix). These figures demonstrate that the proportion of individuals reporting symptoms such as fatigue, headache and cardiopulmonary symptoms has in general been higher among most ethnic minorities than among native Danes during pre-COVID period, i.e., Jan 2018–Dec 2019 (evidence for ethnic disparities). Over time, the proportion of individuals reporting these symptoms decreased from pre-COVID period (Jan 2018–Dec 2019) to COVID period (Jan 2020–Aug 2022) in all ethnic groups, which may be attributed to COVID restrictions put in place during the time of pandemic. However, most ethnic minorities still maintained their higher proportions of reporting these symptoms as compared to the native Danes during pandemic.**

5. Discussion
   *The data is excellent, and the methods are solid, but I think interpretation of what these findings mean is lacking. The discussion is focused primarily on biomedical considerations, but none of these truly explain why ethnic minorities would have increased risk of long COVID. Ethnic minorities were younger, and age was also adjusted for. There was increased hospitalizations and use of ICU, but why? Is there a biologic reason or is the differences in the complex socio-economic, -cultural, - political milieu that they live in that create systemic inequities and ultimately disparities in outcomes. I do not think it is biological and I think onus would be to prove that there are given existing robust evidence on systemic inequities that already present a plausible causal pathway. All of the variable differences discussed potentially have different causal underpinnings.

**Response: We agree with the academic editor that increased risk of long COVID in ethnic minorities may be influenced by differences in socioeconomic conditions, cultural factors, and political factors. Since we have already adjusted for age, sex, civil status, comorbidities, income, and education in our regression models, we have refrained ourselves from discussing in detail about these factors. For example, income and education are part of socioeconomic status, and that is why in this version we discuss other aspects of socioeconomic status such as working and living conditions as other potential contributing factors to the risk of long COVID. We have also removed the discussion about age and comorbidities. We have focused mainly on the factors that we did not adjust for in our models which could have potentially led to the increased risk of long COVID in ethnic minorities including individual-related factors (i.e., poor working and living conditions, cultural beliefs, health seeking behaviors, previous negative health care experience, and mistrust of healthcare system), structural-related factors (i.e., lack of health information in ethnic minorities’ native languages, and absence of professional medical interpreters) and markers of COVID-19 severity (i.e., COVID-19 hospitalization and intensive care use). In summary, we have restructured this section and added the discussion about the mentioned factors in page 15–18 line 358–463.**

*I would highly consider consulting the frameworks and thinking of authors like Camara Jones and Rhea Boyd to gain additional insights for how to conceptualize design and causal considerations when considering disparities. It mostly applies to racism in the United States by a lot of the principles are applicable. The authors already apply thoughtful use of DAGs and I think this could flesh them out more and allow at least discussion of the main causal pathways including why migration may lead to increased hospitalizations.

**Response: Thank you for this useful suggestion. We have restructured our discussion and included several individual- and structural-related factors that may have contributed to the increased risk of long COVID among ethnic minorities living in Denmark. The individual-related factors include poor working and living conditions, cultural beliefs, health seeking behaviours, and mistrust of healthcare system. The structural-related factors include lack of health information in ethnic minorities’ native languages and absence of professional medical interpreters. In addition, we proceeded further to discuss how each factor may have influenced the risk of long COVID in ethnic minorities (see page 15–18 line 358–463). This is to certain extent in line with the institutionalized mediated, and personally mediated levels of racism described by Camara Jones and Rhea Boyd.**

FINANCIAL DISCLOSURE

The funding statement should include: specific grant numbers, initials of authors who received each award, URLs to sponsors’ websites. Also, please state whether any sponsors or funders (other than the named authors) played any role in study design, data collection and analysis, the decision to publish, or preparation of the manuscript. If they had no role in the research, include this sentence: “The funders had no role in study design, data collection and analysis, decision to publish, or preparation of the manuscript.”

**Response: We have described the grant number and initials of who received the fund. In addition, we have stated that “the funders had no role in study design, data collection and analysis, decision to publish, or preparation of the manuscript” in the submission system.**
ABSTRACT
1) Abstract Methods and Findings:
*Please revise the first sentence

**Response: We have rephrased the sentence (page 2 line 35–37). Now it reads “We used nationwide register-based cohort data on individuals diagnosed with COVID-19 aged ≥18 years (n=2 286 955) between January 2020 and August 2022 in Denmark”**

*Please provide brief demographic details of the study population (e.g. sex, age).

**Response: We have provided brief** **demographic details of the study population (page 2 line 40-43).**
*In the last sentence of the Abstract Methods and Findings section, please describe the main limitation(s) of the study's methodology.

**Response: We have described the main limitation of the study's methodology accordingly (page 2–3 line 58–60).**

2) Abstract Conclusions:
*Please change ‘in this population’ to ‘in these populations’
**Response: We have changed ‘in this population’ to ‘in these populations’ (page 3 line 64).**
3) Please ensure that all numbers presented in the abstract are present and identical to numbers presented in the main manuscript text.
**Response: We have cross checked the numbers in the in the abstract are identical to numbers presented in the main manuscript text (see page 2 and page 11–12).**
4) PLOS Medicine requests that main results are quantified with 95% CIs as well as p values.  When reporting p values please report as p<0.001 and where higher as the exact p value p=0.002, for example. For the purposes of transparent data reporting, if not including the aforementioned please clearly state the reasons why not.
**Response: We have reported p values in addition to the 95% CIs both in abstract and the main manuscript as per guideline (see page 2 in abstract and page 12–13 line 281–319 in results).**
5) Please include any important dependent variables that are adjusted for in the analyses.
**Response: We have provided information regarding variables adjusted in the analyses, see in abstract page 2 line 43–44, and statistical analysis, page 10 line 234–236 and line 244–247.**

6) Throughout, suggest reporting statistical information as follows to improve clarity for the reader “22% (95% CI [13%,28%]; p</=)”. Please amend throughout the abstract and main manuscript.
**Response: We have amended the layout of statistical information as per guideline in abstract (page 2 line 45–51) and in results (page 12–13** **line 281–319).**
7) Please note the use of commas to separate upper and lower bounds, as opposed to hyphens as these can be confused with reporting of negative values.

**Response: We have used commas to separate upper and lower bounds (page 2 line 45–51 in abstract and page 12–13 line 281–319 in results).**
8) When a p value is given, please specify the statistical test used to determine it.
**Response: We have specified the statistical test used as per guideline, see page 11 line 254–255.**

INTRODUCTION
1) If possible, include the citations for the relevant studies in this sentence: “Overall, the previous studies have several shortcomings, including studies were based on a single hospital setting or localized area, the studies did not compare symptoms distribution before and after COVID-19 diagnosis, and most of the studies were survey-based.”

**Response: We have added the citations in the manuscript (page 4 line 85–90)**

METHODS AND RESULTS
1) For all observational studies, in the manuscript text, please indicate: (i) the specific hypotheses you intended to test, (ii) the analytical methods by which you planned to test them, (iii) the analyses you actually performed, and (iv) when reported analyses differ from those that were planned, transparent explanations for differences that affect the reliability of the study's results. If a reported analysis was performed based on an interesting but unanticipated pattern in the data, please be clear that the analysis was data-driven.

**Response: We have provided two subsections in the statistical analyses section, namely “analysis plan and amendments” and “main analysis”. More detailed information about the hypotheses tested and analytical plan and amendments is provided on page 9–11 line 207–255.**

2) Did your study have a prospective protocol or analysis plan? Please state this (either way) early in the Methods section.

**Response: The study has a research protocol. We have stated this in page 6 line 136–137­.**
a) If a prospective analysis plan (from your funding proposal, IRB or other ethics committee submission, study protocol, or other planning document written before analyzing the data) was used in designing the study, please include the relevant prospectively written document with your revised manuscript as a Supporting Information file to be published alongside your study, and cite it in the Methods section. A legend for this file should be included at the end of your manuscript.

**Response: We have attached the required protocol associated with ethical approval and data collection from the Danish Health Data Authority as supporting documents (S1 Study Protocol) and cited it accordingly in the methods section (page 6 line 136–137).**

b) If no such document exists, please make sure that the Methods section transparently describes when analyses were planned, and when/why any data-driven changes to analyses took place.

**Response: We have added the document on the protocol of our study (see S1 Study protocol).**
c) In either case, changes in the analysis-- including those made in response to peer review comments-- should be identified as such in the Methods section of the paper, with rationale.

**Response: We have described changes made in the analysis as below, see page 9 line 209–226.**

**“With an increasing number of patients attending long COVID clinics during the pandemic, the hypothesis of this study was that ethnic minorities have an increased risk of long COVID diagnosis than native Danes. We developed analytical plan aiming at testing this hypothesis using nationwide register data in Denmark as there were few studies using registers/electronic medical records. We had initially planned to analyse non-hospitalised individuals only but later we decided to include data on both hospitalised and non-hospitalised individuals after a thorough discussion among the investigators. Regarding long COVID symptoms, our original idea was to examine chances of reporting symptoms such as fatigue, headache, cardiopulmonary symptoms within 4 weeks to 6 months after COVID-19 diagnosis among ethnic minorities compared with native Danes. However, upon reviewing the literature, we realised it would be more beneficial to investigate chances of reporting these symptoms even before COVID-19 diagnosis, and that is why we presented our analysis of symptoms within 6 months before COVID-19 diagnosis, 0 to 4 weeks, and >4 weeks to 6 months after COVID-19 diagnosis. After reviewers’ and editors’ input, we added analysis of risk of long COVID diagnosis by number of doses of COVID-19 vaccine and by three different periods of COVID-19 infection reflecting the prevalence of specific variants in Denmark by region of origin: January 2020–June 2021 (alpha, beta, and gamma variants), July 2021–January 2022 (delta variant), and February 2022–August 2022 (omicron variant).”**

3) PLOS Medicine requests that main results are quantified with 95% CIs as well as p values. When reporting p values please report as p<0.001 and where higher as the exact p value p=0.002, for example. For the purposes of transparent data reporting, if not including the aforementioned please clearly state the reasons why not.
**Response: We have provided absolute numbers, point estimates with confidence intervals and p values in abstract (page 2) and in results (page 12–13 line 281–319).**

4) Please include any important dependent variables that are adjusted for in the analyses.

**Response: We have provided information regarding variables adjusted in the analyses, see in abstract page 2 line 43–44, and statistical analysis, page 10 line 23****4–236 and line 244–247.**
5) Suggest reporting statistical information as detailed above – see under ABSTRACT
**Response: We have included absolute numbers, point estimates with confidence intervals and p values in abstract see page 2 and in results see page 12–13 line 281–319.**

6) Please present numerators and denominators for percentages, at least in the Tables [not necessarily each time they're mentioned].
**Response: We have included data on numerators and denominators in every table where applicable.**
7) Please ensure that the study is reported according to the RECORD guideline and include the completed RECORD checklist as Supporting Information. When completing the checklist, please use section and paragraph numbers, rather than page numbers. Please add the following statement, or similar, to the Methods: "This study is reported as per the Reporting of studies Conducted using Observational Routinely-collected health Data (RECORD) statement (S1 Checklist)."

**Response: We have attached completed RECORD checklist and cited accordingly (see S1 Checklist).**
8) Please define abbreviations at first use (e.g., PCR).

**Response: We have defined PCR as polymerase chain reaction see page 5 line 126.**

9) When referring to ethnicities like 'North African' as a group, please use the plural, e.g., 'North Africans'. Please revise throughout the entire manuscript.

**Response: We have now referred ethnicities using plural form as requested in the entire manuscript.**
10) “Overall, 6479 native Danes and 755 ethnic minorities died within 6 months after COVID-19 diagnosis.” – please add percentages.
**Response: We have included the percentages (page 11 line 263–264).**

DISCUSSION
1) Please present and organize the Discussion as follows: a short, clear summary of the article's findings; what the study adds to existing research and where and why the results may differ from previous research; strengths and limitations of the study; implications and next steps for research, clinical practice, and/or public policy; one-paragraph conclusion. Please remove any subheadings.

**Response: We have restructured the discussion section in accordance with PLOS Medicine guideline, academic editor’ and reviewers’ comments (page 14–20 line 345–507). Subheadings have also been removed.**

2) The discussion in its current form does not seem to be of sufficient depth - please revise and take into account the comments made by the academic editor and reviewers.
**Response: We have restructured the discussion in accordance with academic editor’ and reviewers’ suggestions. We now hope that the discussion has sufficient depth and provides through explanations of our findings (page 14–20 line 345–507).**

TABLES
1) Please define all abbreviations used in the tables (including those in Supporting Information files).

**Response: We have added information about any missing abbreviation in every table where applicable.**
2) Table 1: Please define ‘IQR’. Please add details about how ‘Family income’ was defined.
**Response: We have provided the definition of IQR and family income in Table 1.**
3) Table 3: Please define the asterisk and ‘§’.
**Response: We have defined the mentioned asterisk in Table 3 (which is now Table 4).**

FIGURES
1) For all Figures, please ensure that you have complied with our figures requirements.

**Response: We have ensured that figures are in accordance with the PLOS Medicine requirements.**
2) Please define all abbreviations used in figures (figure, figure legend, figure description, including those in Supporting Information files).
**Response: We have ensured that all abbreviations used in the figures are defined.**

3) Please consider avoiding the use of red and green in order to make your figure more accessible to those with color blindness (including those in Supporting Information files).

**Response: We have ensured to avoid red and green colors in the figures submitted, including those in the supporting information.**
4) Please provide titles and legends for all figures (including those in Supporting Information files).
**Response: We have provided titles and legends for all figures submitted, including those in the supporting information.**
5) Figure 1: Typically, a flowchart goes from top to bottom and the figure seems to be missing important details, e.g., the definition of the ethnic minorities or the cause of death, - please revise and add sufficient details.
**Response: We have revised figure 1 and made changes. Definition of ethnic minorities and cause of death is also provided.**
6) Figure 4: Please see the comment made by Reviewer #2. If you decide to keep the current format, please indicate in the figure caption the meaning of the bars and whiskers.
**Response: We have replaced figure 4 with the results from Table S4 (which is now S10 Table) containing the odds ratios in accordance with reviewer #2. Therefore, a forest plot is presented as figure 4.**

SUPPLEMENTARY MATERIAL
1) Please define all abbreviations used in figures and tables (e.g., ‘ICD’).

**Response: We have defined all abbreviations used in figures and tables.**
3) Figure S1: Please describe the meaning of the colors of the lines and dots.
**Response: We have defined the meaning of the colors of lines and dots in Figure S1.**
4) Table S2: Please see comments for Table 1
**Response: We have provided the definition of IQR and family income in Table S2 (now S2 Table).**

5) Table S3: Please change to “Age groups (years)”.
**Response: We have changed the label from “age groups” to “age groups (years)” in Table S3 (now S5 Table).**
6) Table S6: The asterisk is missing in the description.
**Response: We have removed the asterisk as it has got no description.**

REFERENCES
1) PLOS uses the numbered citation (citation-sequence) method and first six authors, et al.
**Response: We have ensured to write the first six authors in case of more than six as per requirement (****page 21–24 line 508–648).**
2) Please ensure that journal name abbreviations match those found in the National Center for Biotechnology Information (NCBI) databases and are appropriately formatted and capitalized.
**Response: We have ensured that the abbreviations of journal names do match with those found in the NCBI databases (page 21–24 line 508–648).**
3) Where website addresses are cited, please specify the date of access.
**Response: We have specified the website and the date of access (page 21–24 line 508–648).**

4) Please also see for further details on reference formatting.

**Response: Thanks. We have followed the guideline of writing references as per journal requirement (page 21–24 line 508–648).**

**Comments from the reviewers:**

**Reviewer #1:**
1. The study population is defined by the first time someone tested positive, but I wonder what the chances are that someone first testing positive towards the end of the study period had truly never had COVID before? Does this matter to the analysis at all? Also, calendar time in general does not appear to have been taken into account in the analysis in any way. Assuming the different variants have different propensity to lead to long-COVID, would there be any value in looking at the different waves of infections in this analysis? Infection wave, or predominant variant, might come under the group of variables on the causal pathway, and not adjusted for in most analyses, but could be a predictor in secondary analyses.

**Response: We conducted additional analysis showing the risk of long COVID diagnosis in different periods during pandemic, reflecting the prevalence of specific COVID-19 variants in Denmark, see Table 3 included in the main manuscript. Overall, ethnic disparities in the risk of long COVID were more pronounced in the early phase of COVID pandemic as compared to latter phases. We agree with the reviewer that variants/phases of the pandemic could have played a role. In the early phase of the pandemic the health care system also had less knowledge and experience on how to treat the COVID-19 infection leading to more severe consequences. Therefore, we believe that it is not only about the variants but also the development of COVID treatment that has changed over time. We have included this information in abstract (page 2 line 53–54), analysis (page 9 line** **222–226 and page 10 line 243–244), results (****page 13 line 322–324) and discussion (page 18–19 line 447–455). Furthermore, we agree with the reviewer that there is a possibility that some individuals who had COVID-19 infection never had taken a test for COVID-19. However, with massive testing conducted in Denmark, considering testing was free of charge, and accessing to many public places required a negative test, we believe that disparities in testing are more likely to be minor.**

2. There is a sentence at the end of the "Outcomes" section on page 4: "The analysis was restricted to the population experiencing these groups of symptoms within 6 months before COVID-19 diagnosis, 0 to 4 weeks (acute phase of COVID-19 infection), and >4 weeks to 6 months after COVID-19 diagnosis." This confused me - does it mean that people who never experienced these symptoms were excluded from this analysis, or does it mean that this analysis only considers outcomes that occurred within these time windows?
**Response: It means that analysis only considers the specified outcomes that occurred within the mentioned time windows. We have rephrased the sentence and now it reads as follows “We analysed the specified groups of symptoms in three different periods: within 6 months before COVID-19 diagnosis, 0 to 4 weeks (acute phase of COVID-19 infection), and >4 weeks to 6 months after COVID-19 diagnosis.” (page 8 line 186–189).**

3. In the "Covariates" section, it states that age was initially considered as a continuous variable, and then categorised. Was there a particular reason for the categorisation used?

**Response: The reason for creating age groups category was only to tease out if older age was associated with increased risk of long COVID across various ethnic groups. However, in the rest of the analyses, age was treated as a continuous variable.**

4. Subgroup analyses are done by ethnic minority groups and by age, but not by sex. Would that not be worth looking at? Also, when looking at subgroups, it is usually best to use interaction terms within regression models to test for differences in associations. This might help to prevent reading too much into the subgroup analyses, for example, on page 8, the sentence "Further analysis showed that individuals not receiving COVID-19 vaccine exhibited greater risk of long COVID diagnosis than individuals vaccinated; and the association was found in native Danes only (aHR 1.47; 95% CI 1.33-1.63)" - this gives the impression that this association is limited to native Danes, but it could easily be the case that the same association exists in other ethnic groups, but fails to reach statistical significance due to smaller sample size. However, a test of interaction between ethnic group and vaccination status might indicate whether the association is actually different between ethnic groups.

**Response: Thanks for these very useful suggestions. We have therefore performed two additional analyses. The first analysis shows the effect of sex on the risk of long COVID diagnosis across various ethnic group. We found that among Subsaharan Africans, Middle Eastern, Eastern Europeans, and Western Europeans, the risk of long COVID diagnosis was significantly higher in men than in women. However, in the native Danish population, the risk of long COVID diagnosis was significantly lower among men than among women. The second analysis is the stratified analysis exploring the role of ethnicity within men population and women population separately. Our findings show that compared with native Danish men, risk of long COVID diagnosis was higher in men from North Africa, Middle East, Subsaharan Africa, Asia, and Eastern Europe. However, no ethnic differences in long COVID risk among the population of women were observed. The results of the additional analyses are attached in S6 and S7 Tables and cited in the results section (****page 12–13 line 29****8–305).**

**Regarding subgroup analyses we did not do separate analysis for native Danes and ethnic minorities. We created a variable that took into account “all ethnic groups” and “vaccination status (as yes or no)”. Then we adjusted for all relevant confounders as identified by directed acyclic graphs. In conclusion, we got similar results using both approaches i.e., subgroup analysis and interaction term (ethnic minorities*vaccination status) in the regression models. There was no significant difference in the risk of long COVID in ethnic minorities by COVID-19 vaccination status. The association was detected in native Danes only.**

5. In the tables, there is information about the denominators, or the numbers of people testing positive for COVID for the first time, in relation to various factors (Table 1). The tables then move straight on to reporting hazard ratios. I did not see any raw data showing the incidence of long COVID. Could some information be given about the numbers of diagnoses and person-years of follow-up, and event rates, within subgroups of interest? Perhaps something for the supplementary tables.

**Response: Thank you for making this point. We have provided estimates on age standardised incidence rates of long COVID per 100 000 person years among ethnic groups (see S3 Table) and attached raw data as supplementary information (see S2 Appendix). We have updated the text in the methods (page 10 line 229–232) and results section (page 12 line 279–281) accordingly.**

**Reviewer #2:**
**Substantial comments**
1.      Results - Risk of long COVID diagnosis: the main results show that North African, Middle Eastern, Eastern European and Asian ethnicities all had higher hazards of long COVID diagnosis, and as noted these are all groups who were more likely to be hospitalised. Is it possible that the effects seen by ethnicity are actually just effects due to hospitalisation? See comments below for how to investigate this.

**Response: Yes, COVID-19 hospitalisation is indeed driving the association to some extent as presented in Table 2.**
2.      Results - Risk of long COVID diagnosis: in order to try and address the point above I think the authors have performed the analysis included in Table 2, but this only looks at the effect of hospitalisation within each group of ethnicity, it doesn't show whether the overall ethnicity effect observed in the main analysis is due to hospitalisation. I would recommend an additional set of analyses where the main analysis (i.e. figure 2) is repeated within a restricted cohort of people - those who were not hospitalised (i.e. to see if there is still an effect of ethnicity when the effect of hospitalisation is removed). It would also be worth performing this analysis only in the group who were hospitalised.

**Response: We have included the analysis of hospitalised patients and non-hospitalised individuals separately as requested, see S8 Table, and cited in the manuscript. In summary, Eastern Europeans and Middle Eastern still had a higher risk of long COVID diagnosis than native Danes among non-hospitalised individuals. In addition, ethnic disparities were more pronounced in hospitalised patients. We have updated this finding in results (page 13 line 314–316) and in discussion (page 18 line 42****8–431).**
3.      Discussion: the discussion should be updated based upon the results of 2.

**Response: We have updated the discussion about the role of ethnicity on the risk of long COVID after removing the effect of hospitalisation (page 18 line 428–431).**

4.      Discussion: a related point that isn't touched on in the discussion but it would be good to see a comment on is what about the fact that those who were hospitalised are more likely to get a long COVID diagnosis if the clinician knew they were hospitalised, and more ethnic minorities were hospitalised? I am not sure there is much that can be done about this bias but it should be discussed, in conjunction with the new results from 3. above.

**Response: We have discussed that ethnic minorities were more likely than native Danes to be hospitalised for COVID-19 as it is evident in our data (see Table 1). Furthermore, we also related this with their increased risk of long COVID in conjunction with removing the effect of hospitalisation (see in the discussion page 17****–18 line** **421–430).**

5.      Overall thought on substantial comments: it is still important to show and highlight that ethnic disparities continue into long COVID as the authors have done, but there is an opportunity here to tease out the role of prior hospitalisation which has been missed.

**Response: In the current version of manuscript, we have added analysis of non-hospitalised and hospitalised individuals separately (see S8 Table).**
**Minor comments**
1.      Abstract - Background: "infection rates and hospitalisation" - also death from COVID e.g. see Mathur et al

**Response:** **Thank you for this suggestion. We have updated information about death from COVID (page 2 line 31).**
2.      Abstract - Methods and Findings: "in both unadjusted and adjusted models" - I would remove this as its already specified that the results discussed are adjusted

**Response: We have removed “in both unadjusted and adjusted models” and rephrased the statement (page 2 line 43–48).**
3.      Abstract - Methods and Findings: "greater" - should this be "greatest"?

**Response: We have used “greater” since we are reporting several ethnic minority groups compared with native Danes. Our aim was not to report only the most extreme group.**
4.      Introduction: (same comment as abstract): "infection rates, hospitalisation and severe morbidity" - more deaths also reported previously in ethnic minority groups

**Response:** **We have added information about death from COVID and included the reference stated (****page 3 line 78).**
5.      Introduction: "country origin" - I guess this should be "country of origin"

**Response: Thank you. We have corrected “country origin” to “country of origin” (page 5 line 116).**

6.      Introduction: "taking into account…hospitalisation and vaccination status" - I am not sure that the study takes account of either of these currently (see later comments).

**Response: We did not take into account COVID related hospitalisation and vaccination status when investigating the risk of long COVID by different ethnic groups, as these variables were mediating factors in accordance with directed acyclic graphs. But we did report data related to these variables as well as examined their influences in the risk of long COVID.**

7.      Methods - Outcome: "fatigue, headache, cardiopulmonary symptoms" - it would be good to see reasoning/references for why these three types were selected for analysis.

**Response: These are long COVID symptoms and there have been reported in multiple studies referenced in the introduction section in page 3 line 68–73 (Lopez-Leon S in scientific report, Caspersen IH in Eur J Epidemiol, NICE guideline on long COVID definition, see references 2–5).**

8.      Methods - Outcome: "The analysis was restricted to the population experiencing these groups of symptoms" - this wording is quite confusing. The study population has already been defined previously as "all individuals residing in Denmark who had first-time tested positive for SARS-COV-2 (COVID-19 diagnosis) aged 18 years or older from January 1, 2020 to August 31, 2022.". Here is sounds like there is further restriction on the study population but I think what is meant is that these are the outcomes that were studied, please rephrase.

**Response: Thank you for pointing this unclarity out. We have rephrased the statement as requested. It now reads as follows “We analysed the specified groups of symptoms in three different periods: within 6 months before COVID-19 diagnosis, 0 to 4 weeks (acute phase of COVID-19 infection), and >4 weeks to 6 months after COVID-19 diagnosis.” (see page 8 line 186–189). Therefore, no restrictions on the study population were applied.**
9.      Methods - Statistical analysis: "and the risk of long COVID diagnosis" - I think this should be "hazard of long COVID diagnosis"?

**Response: We have corrected the wording from “the risk of long COVID diagnosis” to “hazard of long COVID diagnosis” (see page 10 line 240).**
10.     Methods - Statistical analysis: "covariates like in the Cox models" - "covariates as in the Cox models"?

**Response: Thank you. We have corrected the wording from “covariates like in the Cox models” to “covariates as in the Cox models” (page 10 line 247).**

11.     Methods - Statistical analysis: last 2 sentences beginning "All hazard ratios.." - I wasn't sure how this relates to what is presented in Figure 4 (see also specific comment on Figure 4).

**Response: Since we have replaced the old figure 4 in accordance with reviewer #2 and academic editor’s comments. The new figure 4 is a forest plot containing odds ratios, which means the statement “All hazard ratios (HR) and odds ratios (OR) with their corresponding 95% confidence interval (CI) were presented as unadjusted and adjusted, with native Danes regarded as the reference population” will apply.**

12.     Results - hospital contacts related to long COVID symptoms: The probabilities figure (figure 4) is unclear, I would recommend replacing it with a forest-plot type figure (like Figure 2) containing the results (ORs) in Table S4.

**Response: Thank you for this suggestion. We have replaced the old figure 4 with the new figure 4 containing results from Table S4 (which is now S10 Table). Therefore, a forest plot containing odds ratios is presented as figure 4.**

**Reviewer #3:**
INTRODUCTION

1. Final paragraph - Can you clarify what 'cardiopulmonary symptoms'?

**Response: We have clarified that cardiopulmonary symptoms contain dyspnoea, cough, and chest pain (see page 5 line 120).**

METHODS
2. Setting - can you clarify the duration of free access to testing?

**Response: Testing has been free of charge throughout the COVID-19 pandemic and is still free of charge for all residents as it is financed by general taxes in Denmark (page 5 line 130–132). However, public testing was closed on April 1, 2023.**
3. Data sources and study population - can you clarify what is meant by 'symptoms related to hospital contacts'?

**Response: We have rephrased the statement. It is supposed to be hospital contacts related to symptoms (fatigue, headache, dyspnoea, cough, chest pain, depression, and anxiety) before and after COVID-19 diagnosis, see page 6 line 146–148.**

4. Were the symptoms only recorded in hospitalised patients or were primary care data also included?

**Response: Symptoms were recorded during hospital admissions, outpatient attendance, and attendance at emergency department; see page 6 line 148–149. Primary care data does not contain information on specific symptoms.**

5. Region and country of origin - you state that "Individuals originating outside Denmark and their descendants formed the ethnic minority population. Participants originating and/or born in Denmark (native Danes) constituted the reference group". It is not clear from this statement which group were second generation ethnic minorities born in Denmark classified as. Please clarify.

**Response: We have defined descendants as “those who were born in Denmark from parents with foreign citizenship” (page 7 line 166). This is in accordance with the definition used by Statistics Denmark and the citation is included too.**

6. Outcome - in the definition of the Long Covid diagnosis, 'complications' need to be defined and listed too.

**Response: We have provided the definition of long COVID diagnosis, see page 7 line 174–177.**

7**.** By 'hospital' contacts do you mean hospital admissions, casualty attendance, outpatient attendance or all? This needs classification.

**Response: Hospital contacts included hospital admissions, outpatient attendance, and emergencies. The clarification is now provided in page 6 line 146–149.**

8. It is not clear whether 'anxiety' and 'depression' as 'secondary outcomes' were examined as separate diagnosis or symptoms of long covid.

**Response: We did not examine depression and anxiety as a separate category because we did not have a large enough sample among ethnic minorities. This is clarified in methods; see page 8 line 181–186. Additionally, in every table including analysis of symptoms a legend is also provided to make this clear.**

9. Covariates - The 'length of residency' variable. was it equivalent to age for the control group? please clarify.

**Response: Length of residency was not equivalent to age for the control group. We defined length of residency as time difference in years between date of arrival in Denmark and date of COVID-19 diagnosis (page 8–9 line 204–206)**.

RESULTS
10. Participants characteristics - in the sentence 'overall 6479 native Danes and 755 ethnic minorities died within six months' please modify to 'people from ethnic minorities'.

**Response: We have modified the statement to 'people from ethnic minorities' as requested (page 11 line 264).**
11. Risk of long COVID diagnosis - it would be helpful to include absolute numbers besides the hazard ratio and the confidence interval particularly when looking at risk by country of origin to give the reader an idea about the sample size for each category. I can see it's included in figure 3 but it would be good to include in the text too.

**Response: We have inserted absolute numbers in hazard ratios estimates where relevant see page 12–13 line 281–293, line 296, and line 309–314.**

12. In the second paragraph of this section it is not entirely clear what the hazard ratio for those from sub-Saharan Africa aged more than 60 years is in comparison to. This needs clarification in the text.

**Response: The comparison group was native Danes aged more than 60 years. We have provided more clarification see page 12 line 295.**

13. In the DAG (Figure S1), Why is the outcome healthcare contact rather than long COVID diagnosis?

**Response: It was supposed to be long COVID, and we have corrected this see S1 Fig, thanks!**

14. Was there a difference in risk between 'immigrants' and 'descendants' as classified in table 1? did you conduct any subgroup analysis to look at that? it would be helpful to see that if we were going to hypothesise about the causes of the increased risk and ethnic minorities.

**Response: The number of descendants with long COVID diagnosis (n=120) was too small to be grouped by region of origin. That is why we did not proceed further to conduct the analysis. This is included as a limitation of the study (page 20 line 489–490).**

15. In table S2 some of the variables had a category of 'missing' while others did not. Does that mean those with no missing category did not have any missing observations at all?

**Response: In the Danish setting, individuals can easily be tracked via social security number from one register to another. Therefore, we had few missing observations as specified in Table 1 and Table S2 (now S2 Table). However, for 5 missing observations and below, the numbers were aggregated in accordance with Danish Data Protection Act on reporting few observations (see:** [**https://www.dst.dk/Site/Dst/SingleFiles/GetArchiveFile.aspx?fi=3477468153&fo=0&ext=forskning**](https://www.dst.dk/Site/Dst/SingleFiles/GetArchiveFile.aspx?fi=3477468153&fo=0&ext=forskning)**)**
16. From Table S2, it seems some of the most striking differences in vaccination status were for three doses. did you conduct analysis by the number of doses of vaccination?

**Response: We have provided additional analyses showing the risk of long COVID diagnosis by number of doses of COVID-19 vaccine (see S9 Table). The findings show that native Danes only had significantly reduced risk of long COVID diagnosis with receiving two and three doses of COVID-19 vaccine as compared to not receiving vaccination (page 13 line 320–322). Ethnic minorities did not seem to benefit from the vaccination in terms of long COVID risk (page 19 line 460–463).**
17. It is not clear to me how the analysis was conducted for the results in table S3. Was age entered in the model as an independent variable? Did you conduct two subgroup models for the different age groups? please clarify.

**Response: In Table S3 (now S5 Table), we conducted a subgroup analysis where we created a variable which contained information on “all ethnic groups” and “age group as binary variable (18–60 years and >60 years)” as described in methods section (page 10 line 240–242). Hence, we had the following groups: Danes aged 18–60 years, Danes aged >60 years, North Africans aged 18–60 years, North Africans aged >60 years and the same age groups for other ethnic minorities. We then assigned Danes aged 18–60 years as the reference group and adjusted for all other relevant confounders with the purpose of investigating the effect of age group in the risk of long COVID across various ethnic groups. In addition, we have also provided analysis where we look at effect of ethnicity in the risk of long COVID in individuals aged ≤60 years and also effect of ethnicity in the risk of long COVID in individuals aged >60 years as two separate analyses (see S4 Table). We wanted give readers understanding of both perspectives.**
18. Figure 2 - state 'northern Europe other than Denmark' for this category.

**Response: We have stated Northern Europe other than Denmark in the legend and in every Table and Figure where applicable.**

19. More information about the analysis informing the results in Figure 4 are needed. What is the comparison group here?

**Response: We have replaced the old figure 4 containing adjusted proportions with the new figure 4 containing adjusted odds ratios from S10 Table (previously S4 Table) in accordance with academic editor’s and reviewer #2 comments. The comparison group is the 6 months before COVID-19 diagnosis which is stated in the legend text. The new figure 4 is the forest plot.**

20. More information is needed about the analysis comparing the period before COVID diagnosis and after in terms of symptoms. When comparing the symptoms after COVID diagnosis did you adjust for baseline symptoms before COVID-19 diagnosis when comparing between the ethnic group categories? Some of the ethnic group categories had higher baseline risk of the symptoms.

**Response: This is an important point. When reporting analysis of symptoms (for example in S10 Table and the new Figure 4) we have stated that we adjusted for baseline comorbidities using Charlson comorbidity index (CCI). The CCI encompasses 17 health conditions in a period of 5 years prior to the diagnosis of COVID-19. Using CCI provides a greater scope of understanding the health status of individuals prior to COVID-19 diagnosis than the specific symptoms (see page 8 line 195–199).**

DISCUSSION
21. You discuss potential explanations for the observed increased risk of long COVID in ethnic minority groups. However some of these could have been adjusted for in the model as mediators to see if they partially explain the observed significant associations.

**Response: In our models, we only adjusted for confounders (i.e., not mediators) in accordance with directed acyclic graphs which are used as a guide to assess which factors are supposed to be adjusted in the models (see S1 Fig). We only adjusted for age, sex, civil status, comorbidities, income, and education in our regression models. Factors such as COVID-19 hospitalisation and COVID-19 vaccination were mediators and that is why they were not adjusted in the models. However, we performed stratified analysis for some mediating factors (COVID-19 hospitalisation and COVID-19 vaccination) to show their influences in the association (see S8 and S9 Tables).**

22. I think the discussion around hospitalisation and age as risk factors confuses the narrative of the paper a little bit. These factors are established factors in the severity of covid, and also there is emerging evidence of a link with long COVID. The important thing in this analysis is that these factors are accounted for when testing the relationship of ethnicity with long covid rather than confusing that narrative with multiple subgroup analysis.

**Response: Thank you for this observation. We have removed the discussion around age and comorbidities as they are already adjusted for in our models. However, we did not adjust for COVID-19 related hospitalisation as this factor was not a “true confounder” based on the directed acyclic graphs (see S1 Fig). COVID-19 hospitalisation was a mediating factor in the association between ethnicity and the risk of long COVID. That is why we examined the influence of COVID hospitalisation and included this factor in the discussion. In this revised version, we have focused mainly on the factors that we did not adjust for in our models which could have potentially led to the increased risk of long COVID in ethnic minorities, including individual-related factors (i.e., poor working and living conditions, cultural beliefs, health seeking behaviors, previous negative health care experience, and mistrust of healthcare system), structural-related factors (i.e., lack of health information in ethnic minorities’ native languages, and absence of professional medical interpreters) and markers of COVID-19 severity (i.e., COVID-19 hospitalisation and intensive care use). In summary, we have restructured this section and added the discussion about the mentioned factors in page 15–18 line 358–463.**

23. In terms of the discussion around intensive care being a confounder in the relationship why was this not examined? Was this data not available?

**Response: We did not examine the association between intensive care use and the risk of long COVID diagnosis by region of origin because we found a few observations among some ethnic minority groups.**
24. Again the discussion around comorbidities being a possible explanation of the increased risk in ethnic minority groups doesn't need to be speculative as these were included in the models.

**Response: Thanks! We have also removed the discussion about comorbidities as it is already adjusted for in our models. In this revised version, we have focused mainly on the factors that we did not adjust for in our models which could have potentially led to the increased risk of long COVID in ethnic minorities, including individual-related factors (i.e., poor working and living conditions, cultural beliefs, health seeking behaviors, previous negative health care experience, and mistrust of healthcare system), structural-related factors (i.e., lack of health information in ethnic minorities’ native languages, and absence of professional medical interpreters) and markers of COVID-19 severity (i.e., COVID-19 hospitalisation and intensive care use). In summary, the discussion is restructured to reflect these changes (see page 15–18 line 358–463).**
25. The authors then suggest biological variation as a possible explanation after adjusting for comorbidities. For example they mention 'immunological factors' and 'circulatory system'. However the most likely explanation is that there is residual confounding in terms of socioeconomic disadvantage. This needs discussion. This includes a discussion around the completeness, the quality and validity of the data that's assessing socio economic disadvantage in this study.

**Response: We agree this is an important point. The increased risk of long COVID in ethnic minorities may be influenced by socioeconomic disadvantage. Since we have already adjusted for family income and education in our regression models, we have refrained ourselves from discussing in detail about these factors. For example, income and education are part of socioeconomic status, and that is why in this version we discuss other aspects of socioeconomic status such as working and living conditions as other potential contributing factors to the risk of long COVID. Overall, we have taken a broader approach to discussing the factors that may have influenced the association using Andersen’s and Levesque’s models of access to care. We have discussed the factors using the following groups: individual-related factors (i.e., poor working and living conditions, cultural beliefs, health seeking behaviors, previous negative health care experience, and mistrust of healthcare system), structural-related factors (i.e., lack of health information in ethnic minorities’ native languages, and absence of professional medical interpreters) and markers of COVID-19 severity (i.e., COVID-19 hospitalization and intensive care use). The discussion section is restructured to incorporate the suggested changes (see page 15–18 line 358–463). Furthermore, we have provided the discussion about completeness and quality of the data (see page 19–20 line 467–490).**
26. The evidence around the prevalence of long COVID in ethnic minorities is not necessarily consistent. For example in the UK the data from the office for National Statistics does not show higher prevalence of long COVID among ethnic minorities and lower prevalence in some of these groups. The discussion can include potential reasons for the inconsistency of the evidence.

**Response: We have referenced several studies conducted in the UK (e.g., Subramanian et al Nat Med and Thompson et al Nat Comm), which have demonstrated a higher risk of long COVID in people belonging to African and Asian ethnicity. We feel it is more reasonable to discuss the risk of long COVID observed in our study versus the risk of long COVID reported in the other studies (e.g., UK, US, and Netherlands) rather than comparing risk of long COVID versus prevalence of long COVID, in which these two measures of occurrence are calculated based on different denominators. In addition, we believe that migration policies impact the composition of migrants/migrant groups in different countries and that living conditions and socioeconomic circumstances also vary between country contexts. This makes it difficult to compare results between countries. Thus, this is included when discussing the generalizability of our findings (see page 20–21 line 495–500)**

27. Also there needs to be a discussion around the outcome assessment and the context for that. How is long covid diagnosis reached in Denmark? Could healthcare utilisation patterns and behaviours partially influence who gets the diagnosis?

**Response: We have now added more discussion about the registration of long COVID diagnosis in terms of when it started, which hospital contacts are associated with the diagnosis, limitation, and bias around registration of the diagnosis (see page 20 line 474–490). Regarding patterns of healthcare utilisation, we have provided additional analysis on hospital contacts and general practitioner contacts before COVID pandemic (see S1 Appendix). We have also discussed the findings in relation to the risk of long COVID (see page 16 line 389–399).**

28. In the discussion you mention the contrast of clinical based diagnosis versus symptom based diagnosis. It is not very clear what this means. The long COVID diagnosis should be primarily based on symptoms as there is no biomarker or diagnostic imaging for it at the moment.

**Response: We agree with the reviewer that long COVID diagnosis is a symptom-based diagnosis, and it is assigned a specific ICD code in the Danish registers. The contrast was that long COVID diagnosis does share some similarities in terms of symptoms with other established differential diagnoses. This means that doctors may consider other diagnoses first before deciding a long COVID diagnosis. We have rephrased the statement to make this clear page 20 line 480–482.**

29. In your discussion of the implications of these findings, please mention aspects of prevention as a means of reducing health inequalities.

**Response: We have inserted the following statement as aspect of prevention “a concerted effort to address the impact of long COVID among ethnic minorities is needed, particularly focusing on reducing disparities in living and working conditions, advocacy activities for COVID-19 vaccines, and adoption or continuation of preventive measures such as avoiding close contact with confirmed cases of COVID-19 as well as hand washing and use of sanitizers” (page 21 line 509–5013)**

**We have updated tables, figures, and reference numbers accordingly.**

**Best wishes**

**George Frederick Mkoma (on behalf of all authors)**
