# Supplementary material for: Risk of long COVID and associated symptoms after acute SARS-COV-2 infection in ethnic minorities: A nationwide register-linked cohort study in Denmark
Source: PLoS Med. 2024 Feb 20;21(2):e1004280. doi: 10.1371/journal.pmed.1004280 (PMC10914299; doi:10.1371/journal.pmed.1004280)
Supplement: Attachment — Submitted filename: Response to PLOS Medicine_R3_final.docx [file pmed.1004280.s020.docx]

**Response to Comments on Manuscript ID:** PMEDICINE-D-23-02370R2, entitled "Risk of long COVID and associated symptoms after acute SARS-COV-2 infection in ethnic minorities: a nationwide register-linked cohort study in Denmark" by Mkoma GF et al.

Alexandra Schaefer, PhD
Associate Editor
PLOS Medicine

Thank you for giving us the opportunity to submit a revised draft of our manuscript for consideration for publication in *PLOS Medicine*. We appreciate the time and effort that editors and reviewers have dedicated to providing valuable feedback on our manuscript. Below we address each comment point-by-point and provide the exact page number and/or line number where each revision was made, referencing the marked version of the revised manuscript.

**Requests from Editors:**

ACADEMIC EDITOR COMMENTS

1) Clarity on the outcome is needed: I think they need to be clear that that the outcome was an ICD-10 diagnosis of long COVID. Per the ICD-10 definitions, this is supposed to mean complications persisting beyond acute illness. But it is key to recognize that just because a clinician records an ICD-10 diagnosis, it doesn't mean that they did their due diligence. For clarity, I would write that the outcome was ICD-10 diagnosis of long COVID, and then say this is supposed to indicate a scenario where complications with no other explanations exist. Discussing it in this manner at least acknowledges that there may be a discrepancy.

**Response:** **We have rephrased the text describing the outcome of interest in the manuscript. The statement reads as follows “The primary outcome of interest was ICD-10 diagnosis of long COVID identified by ICD-10 codes (B94.8 or B94.8A); and this indicates complications persisting beyond the acute COVID-19 infection that cannot be explained by an alternative diagnosis. The complications encompass symptoms such as** **fatigue, headache, dyspnoea, chest pain, cough, and depression or anxiety that generally have an impact on everyday functioning and may present as new onset following initial recovery from an acute COVID-19 episode or persist from the initial illness” (see line 209-215).**

2) I also still think that Fig S2 strongly suggests that there may be an underlying etiology of disparities in symptomatology (and therefore long COVID diagnosis) that is unrelated to any biologic reasons. I appreciate that in Table 4 the magnitude of some but certainly not all the odds ratios are greater in ethnic minorities. This could easily be by chance and there is no formal statistical assessment to see if disparities were indeed greater in magnitude during the COVID time periods. In several analyses, the point estimate is greater, but confidence intervals overlap. The authors do discuss this in their discussion, but I still think there is an overinterpretation of these findings. I think these points need to be clearer. Although it is possible that these findings indicate an increase in long COVID, it may also be entirely true that there are no real differences (perhaps only differences in reporting and recorded diagnoses). Even if differences are real, they are likely more subtle than most HRs would suggest. For example, taking a difference-in-difference type approach to the Table 4 results would show perhaps only small increases in the disparities when taking into account the disparities that already existed. I know this goes against the author's interpretation but I think needs to be taken into account.

**Response: For clarification, in the analysis presented in Table 4, we had taken into account 17 pre-existing health conditions (using Charlson comorbidity index), age, sex, family income, education, and civil status. We agree with the academic editor that the point estimates of a single long COVID-related symptom such as fatigue or headache were mostly not different among the ethnic groups during different COVID periods due to overlapping confidence intervals in Table 4. As such we have provided a statement that a caution is needed when interpreting these findings of fatigue and headache among ethnic groups (see line 619-622).**

**However, in the same Table 4, we see clearly that there are huge differences in the point estimates of cardiopulmonary symptoms together with their confidence intervals in the majority of ethnic minority populations, especially in the period beyond 4 weeks up to 6 months after COVID-19 diagnosis as compared to 6 months before COVID-19 diagnosis which indicate the long COVID issue. We therefore believe that these differences may be true for cardiopulmonary symptoms and may be small or non-existent for fatigue and headache among ethnic groups compared. Consequently, it is possible that the greater hazard ratios of ICD-10 long COVID diagnosis reported in some ethnic minorities were largely influenced by cardiopulmonary symptoms.**

**Furthermore, we agree with the academic editor that taking into account pre-existing disparities is an important issue, which is why we included 17 pre-existing health conditions (using Charlson comorbidity index) when estimating the risk of long COVID symptoms (see footnotes in Table 4). However, we acknowledge that we did not take into account some other aspects of disparities, which are connected to culture, behavioural factors, healthcare providers attitude and stereotypes, or mistrust of healthcare system as such data were unavailable (see line 622-626 in the limitation).**

EDITORIAL COMMENTS

1) Methods/Limitations: Please discuss in more detail whether you considered a single long COVID-related symptom as “Long COVID”. Please discuss whether there is a possibility that symptoms might be unrelated to COVID/long COVID. In this regard, we also feel it is important that you emphasize the low numbers in some groups (e.g., consultations for individual symptoms; table 4) in the limitations.

**Response: In the current version of the manuscript, we have discussed this issue as a limitation of the study as follows: “Another limitation was that we considered a single long COVID-related symptom such as fatigue or headache as ‘long COVID’ as per NICE guidelines stating that any sign and symptom that continue or develop after acute COVID‑19 infection (from 4 weeks or more) are considered long COVID if not explained by an alternative diagnosis. Although we adjusted for 17 pre-existing health conditions using Charlson comorbidity index when estimating odds ratios of reporting long COVID symptoms, there is still a possibility that these symptoms might be unrelated to COVID-19/long COVID and hence this may have resulted in bias on the estimates reported. Owing to a few observations in the descendant group, we could not explore intergenerational differences in the risk of long COVID. In addition, due to low count in some ethnic groups, we could not perform analysis of a single symptom by largest countries of origin.” (see line 606-616)**

2) Limitations: Please discuss the influence on medications/treatment on your study design/results and mention whether these data were available in the registries and, if yes, why you chose not to incorporate these data into this study.

**Response:** **Data on medication used at hospitals are currently not available in the Danish registers; and that is why we could not investigate the influence of the medication in the risk of long COVID comparing ethnic minorities and native Danes. We have described this as a limitation of our study (see line 616-619).**

GENERAL

1) Please carefully review the terms used to describe ethnic minorities in your study. For example, "Iraqis" does not seem appropriate because the groups are a combination of immigrants and their first-generation descendants. We suggest using, for example, "people of Iraqi origin" or, e.g., “people of North African origin" (as you have already done on occasion). Please make sure these terms are used consistently throughout the paper.

**Response: Thank you for the suggestion. We have rephrased the wording of all ethnic groups in the entire manuscript where applicable. For example, Iraqis to people of Iraqi origin, Turks to people of Turkish origin, North Africans to people of North African origin and so on.**

2) It seems that line numbering has only been added to the marked-up version of your manuscript. Please check.

**Response: We have added line numbering to the clean version of manuscript too.**

ABSTRACT

1) Line 45: Please define ‘CI‘ at first use.

**Response: We have defined confidence interval (CI) in the abstract (see line 45).**

AUTHOR SUMMARY

The Author summary in its current form is rather long, particularly ‘Why was this study done?’ and ‘What do these findings mean?’. Please try to shorten the individual bullet point and to focus on core details.

**Response: We have shortened the author summary, and the current word count is 288 from 354 words (see page 3).**

INTRODUCTION

l.130: The terms gender and sex are not interchangeable (as discussed in <https://eur02.safelinks.protection.outlook.com/?url=https%3A%2F%2Fwww.who.int%2Fhealth-topics%2Fgender&data=05%7C01%7Cgeorge.mkoma%40sund.ku.dk%7C37f243e40e234d7c67e708dbf2488fbe%7Ca3927f91cda14696af898c9f1ceffa91%7C0%7C0%7C638370165684134974%7CUnknown%7CTWFpbGZsb3d8eyJWIjoiMC4wLjAwMDAiLCJQIjoiV2luMzIiLCJBTiI6Ik1haWwiLCJXVCI6Mn0%3D%7C3000%7C%7C%7C&sdata=8EDS47BZ0UzsWjoZFfJ2SL5kALpnLhhvrlPkYg3EiZ8%3D&reserved=0>); please be sure to use the appropriate term.

**Response: We have used the term “sex” instead of “gender” (see line 143).**

DISCUSSION

ll.518-519: „Living in these circumstances may sometimes be culturally influenced but can have detrimental effect in individual’s recovery from COVID-19 infection.” – please provide a reference.

**Response: We have inserted the reference (see line 517-518).**

FIGURES

Figure 1: For easy comparison, we suggest adding percentages in the boxes.

**Response: We have now included the percentages in Figure 1 (page 14).**

TABLES

S12 Table: Please add a reference/footnote to S12 Table so that the reader can easily find the denominators used for the calculations in this table.

**Response: We have inserted the denominators in S12 Table to provide clarity**.

REFERENCES

Please use ‘accessed’ instead of ‘cited’ when specifying the date of access.

**Response: We have now used the word “accessed” instead “cited” in the references (pages 29-31).**

SOCIAL MEDIA

To help us extend the reach of your research, please provide any X (formerly known as Twitter) handle(s) that would be appropriate to tag, including your own, your coauthors’, your institution, funder, or lab. Please respond to this email with any handles you wish to be included when we tweet this paper.

**Response: We have provided twitter handles in the email (@mkomageorge, @Agyemang_c).**

**Comments from Reviewers:**

Reviewer #1: Alex McConnachie, Statistical Review

I thank the authors for their consideration of my original comments. All of their responses are good, and I have no further comments.

**Response: Thank you!**

Reviewer #2: Thanks, all comments addressed well

**Response: Thank you!**

Reviewer #3:

1. Setting - can you clarify the duration of free access to testing? The authors responded: Testing has been free of charge throughout the COVID-19 pandemic and is still free of charge for all residents as it is financed by general taxes in Denmark (page 5 line 130-132). However, public testing was closed on April 1, 2023." - though I could only find a statement about access to care being free of charge on page 5. Could they add the specific response about testing to the text?

**Response: We have incorporated information about testing for COVID-19 infection being free of charge in Denmark in the text (see line 141-142 in the introduction).**

2. Region and country of origin - you state that "Individuals originating outside Denmark and their descendants formed the ethnic minority population. Participants originating and/or born in Denmark (native Danes) constituted the reference group". It is not clear from this statement which group were second generation ethnic minorities born in Denmark classified as. Please clarify. The authors responded stating the definition of descendants as "those who were born in Denmark from parents with foreign citizenship" but my point was that these overlap with the reference group of born in Denmark" - please clarify.

**Response: As per Statistics Denmark definition, descendants of ethnic Danes and descendants of ethnic minorities are never classified into the same group. These two groups have different coding system based on the data from Statistics Denmark and can explicitly be separated from one another. Therefore, in our study, the same approach was used to define the groups i.e., Danes and their descendants (native Danes) and immigrants and their descendants (ethnic minorities) (see line 202-205).**

3. Outcome - in the definition of the Long Covid diagnosis, 'complications' need to be defined and listed too. This has also not been adequately addressed by the authors. Is the correct word here "symptoms" rather than "complications"?

**Response: We have rephrased the text in the outcome to provide clarity. The statement reads as follows: “The primary outcome of interest was ICD-10 diagnosis of long COVID identified by ICD-10 codes (B94.8 or B94.8A); and this indicates complications persisting beyond the acute COVID-19 infection that cannot be explained by alternative diagnosis. The complications encompass symptoms such as fatigue, headache, dyspnoea, chest pain, cough, and depression or anxiety that generally have an impact on everyday functioning and may present as new onset following initial recovery from an acute COVID-19 episode or persist from the initial illness” as per ICD-10 definition (see line 209-215).**

4. You discuss potential explanations for the observed increased risk of long COVID in ethnic minority groups. However some of these could have been adjusted for in the model as mediators to see if they partially explain the observed significant associations -The authors state that they did not adjust for medication but did not offer a justification for this. DAGs don't prevent the adjustment for mediation for the direct effect of the exposure on the outcome.

**Response: In our previous reply this is how we responded, in our models, we only adjusted for confounders (i.e., not mediators) in accordance with directed acyclic graphs which are used as a guide to assess which factors are supposed to be adjusted in the models. However, we agree with the reviewer that medication is an important factor which should have been considered in the models. In the current version of manuscript, we have addressed this as follows: “Data on medication used at hospitals are currently not available in the Danish registers; and that is why we could not investigate the influence of medication in the risk of long COVID comparing ethnic minorities and native Danes.” We have described this as a limitation of our study (see line 616-619).**

5. In your discussion of the implications of these findings, please mention aspects of prevention as a means of reducing health inequalities - the authors have added a statement about avoiding close contact with cases, handwashing and use of sanitizers, though the last two are not the main preventive measures for covid given its airborne transmission: <https://eur02.safelinks.protection.outlook.com/?url=https%3A%2F%2Fwww.who.int%2Fnews-room%2Fquestions-and-answers%2Fitem%2Fcoronavirus-disease-covid-19-how-is-it-transmitted&data=05%7C01%7Cgeorge.mkoma%40sund.ku.dk%7C37f243e40e234d7c67e708dbf2488fbe%7Ca3927f91cda14696af898c9f1ceffa91%7C0%7C0%7C638370165684134974%7CUnknown%7CTWFpbGZsb3d8eyJWIjoiMC4wLjAwMDAiLCJQIjoiV2luMzIiLCJBTiI6Ik1haWwiLCJXVCI6Mn0%3D%7C3000%7C%7C%7C&sdata=EnMAHhir%2FRA4bK3HikNxzyDb3ufVjmsvOLQBmA3BnFk%3D&reserved=0>.

**Response: Thank you for the suggestion. We have rephrased the text and written the following statement: “To decrease health inequalities, we must address a variety of public policy issues, combining initiatives that target economic and social injustices with targeted attention to marginalized communities and groups such as ethnic minorities. This could entail using legislation, taxation, regulation, and policy to ensure a more equitable distribution of wealth, power, and income. To address the impact of long COVID among ethnic minorities a concerted effort is needed, particularly focusing on equal access to high-quality housing and improved working conditions for everyone, advocacy activities for COVID-19 vaccines, and adoption or continuation of preventive measures such as avoiding close contact with confirmed cases of COVID-19 as well as handwashing and use of sanitizers.” (see line 641-650).**

**With regard to COVID-19 transmission, we stated about handwashing and use of sanitizers because the COVID-19 infection can spread from one person to another through various ways in accordance with WHO and CDC. In addition, these strategies are also recommended as part of long COVID prevention by WHO/CDC.**

1. **Current evidence suggests that the virus spreads mainly between people who are in close contact with each other, for example at a conversational distance. The virus can spread from an infected person’s mouth or nose in small liquid particles when they cough, sneeze, speak, sing or breathe (this is often called short-range aerosol or short-range airborne transmission) or if infectious particles come into direct contact with the eyes, nose, or mouth (droplet transmission).**
2. **The virus can also spread in poorly ventilated and/or crowded indoor settings, where people tend to spend longer periods of time.**
3. **People may also become infected when touching their eyes, nose, or mouth after touching surfaces or objects that have been contaminated by the virus.**

**Best regards**

**George Frederick Mkoma (on behalf of all authors)**
