# Supplementary material for: Risk of long COVID and associated symptoms after acute SARS-COV-2 infection in ethnic minorities: A nationwide register-linked cohort study in Denmark
Source: PLoS Med. 2024 Feb 20;21(2):e1004280. doi: 10.1371/journal.pmed.1004280 (PMC10914299; doi:10.1371/journal.pmed.1004280)
Supplement: Attachment — Submitted filename: Response to PLOS Medicine_R4.docx [file pmed.1004280.s021.docx]

**Response to Comments on Manuscript ID:** PMEDICINE-D-23-02370R3, entitled "Risk of long COVID and associated symptoms after acute SARS-COV-2 infection in ethnic minorities: a nationwide register-linked cohort study in Denmark" by Mkoma GF et al.

Alexandra Schaefer, PhD
Associate Editor
PLOS Medicine

Once again, thank you for giving us the opportunity to submit a revised draft of our manuscript for consideration for publication in *PLOS Medicine*. We appreciate the time and effort that editors and reviewer(s) have dedicated to providing feedback. Below we address each comment point-by-point and provide the exact page number and/or line number where each revision was made, referencing the marked version of the revised manuscript.

**Requests from Editors:**

1) Please revise your Author summary with an emphasis on using non-technical language. It is better to avoid words like " significantly " and please avoid reporting data/exact values ("1.2 to 1.4 times higher risk").

**Response: In the current Author summary, we have removed technical terms (e.g., significantly) and we have also avoided reporting numbers/data, see page 3.**

2) Under ‘What did the researchers do and find?’, please use non-technical language to describe your findings. Editorial suggestion: People of North African, Middle Eastern, Eastern European, and Asian origin were more likely to report cardiopulmonary symptoms (including dyspnea, cough, and chest pain) and any long COVID symptoms than native Danes, especially beyond 4 weeks to 6 months after COVID-19 diagnosis.

**Response: Thank you for the suggestion. We have therefore inserted the text “People of North African, Middle Eastern, Eastern European, and Asian origin were more likely to report cardiopulmonary symptoms (including dyspnea, cough, and chest pain) and any long COVID symptoms than native Danes, especially beyond 4 weeks to 6 months after COVID-19” diagnosis.” (page 3, line 84–87).**

3) ll.592-595: Please provide reference (for the NICE guidelines; reference 5?)

**Response: We have inserted reference 5, see line 584–587.**

4) Figure 1: For consistency, change "Danes" to "Native Danes" and add the definition of "Native Danes" in the figure description.

**Response: We have changed the word from Danes to Native Danes in Figure 1. We have also defined “Native Danes” in the figure description, see page 13 and line 343.**

5) S2 Figure: We suggest that you start the axis of all four graphs at zero. If this is not possible, please show a break in the axis. Also, please include a note in the figure description stating that the y-axis is not identical for the four graphs. Please add a label for the x-axis, such as "time frame" or "time period".

**Response: We have corrected the S2 Figure by starting the axis of all four graphs at zero. In addition, we have inserted the label “time period” in each x-axis of the four graphs, see the new S2 Figure.**

6) S1 Appendix: This one has no reference in the main manuscript and does not have a title nor a description. Please revise.

**Response: Thank you for noting this! We have now cited S1 Appendix in the main manuscript (line 267–270). Additionally, we have included the title and description of the data submitted in the list of legends (line 859).**

Comments from Reviewers #3:

1) Please include the statement in your response in the text of the paper to make this clear to international audiences: "As per Statistics Denmark definition, descendants of ethnic Danes and descendants of ethnic minorities are never classified into the same group. These two groups have different coding system based on the data from Statistics Denmark and can explicitly be separated from one another."

**Response: We have included the statement in the methods “As per Statistics Denmark definition, descendants of ethnic Danes and descendants of ethnic minorities are never classified into the same group. These two groups have different coding system based on the data from Statistics Denmark and can explicitly be separated from one another." (line 204****–207).**

2) My comment from R2: "You discuss potential explanations for the observed increased risk of long COVID in ethnic minority groups. However, some of these could have been adjusted for in the model as mediators to see if they partially explain the observed significant associations -The authors state that they did not adjust for medication but did not offer a justification for this. DAGs don't prevent the adjustment for mediation for the direct effect of the exposure on the outcome." - I am sorry there was a typo in my review. What I meant was 'mediation' not 'medication' - I was requested you justify why you haven't adjusted for mediation. Please do so.

**Response: The reason that we did not adjust for mediation or mediators was because mediators are deemed to belong in the causal pathway between the exposure (migration) and outcome of interest (long COVID). Hence, adjusting for mediators would have induced bias as suggested in the Tutorial on Directed Acyclic Graphs (DAG). We provided this clarification and have additionally added the citation on the Tutorial on DAG, see line 27****4–278. Furthermore, to show the influence/effect of mediators e.g., COVID-19 hospitalisation or COVID-19 vaccination on long COVID, the approach was to provide stratified analysis rather than adjustment in the models e.g., S8 Table.**

3) In your discussion of the implications of these findings, please mention aspects of prevention as a means of reducing health inequalities - the authors have added a statement about avoiding close contact with cases, handwashing and use of sanitizers, though the last two are not the main preventive measures for covid given its airborne transmission:

I am afraid this comment has not been adequately addressed as the preventive methods listed stayed the same from the previous version however the authors acknowledge airborne transmission in their response. Please add preventive measures related to airborne transmission to the text.

**Response: In the current version of manuscript, we have included social distancing, avoiding poorly ventilated areas, and use of face masks in crowded environments as preventive measures to reduce the chances of COVID airborne transmission (line 626–627).**

**We have also updated the reference list and cited them accordingly as per PLOS Medicine guideline.**

With best regards

George Frederick Mkoma (on behalf of all authors)
